# Clinical Outcomes of Pandrug-Resistant Versus Carbapenem-Resistant, Colistin-Susceptible *Acinetobacter baumannii* Infections: A Retrospective Analysis of a Prospective Multicentre Cohort

**DOI:** 10.3390/antibiotics15080809

**Published:** 2026-08-19

**Authors:** Ilias Karaiskos, George L. Daikos, Sofia Michelidou, Christina Mouratidou, Alexandra Gavala, Aikaterini Gkoufa, Aikaterini Sakagianni, Eleni Mouloudi, Evdoxia Tsigou, Christina Routsi, Stamatis Karakonstantis, Christina Stamatopoulou, Despina Markantonaki, Foteini Veroniki, Maria Pirounaki, Anna Kyriakoudi, Sevasti Ampelioti, Charalambos Anastogiannis, Antonia Koutsoukou, Helen Giamarellou, Konstantinos Pontikis

**Affiliations:** 1 Hygeia General Hospital, 1st Department of Internal Medicine, Infectious Diseases, 15123 Athens, Greece; ikaraiskos@hygeia.gr (I.K.); e.giamarellou@hygeia.gr (H.G.); 2 Mitera General Hospital, 2nd Department of Internal Medicine, 15123 Athens, Greece; 3 Sismanogleio General Hospital of Attica, Intensive Care Unit, 15126 Athens, Greece; 4 Hippokration General Hospital of Thessaloniki, Intensive Care Unit, 54642 Thessaloniki, Greece; 5 General and Oncologic Hospital of Kifisia ‘Oi Agioi Anargyroi’, Intensive Care Unit, 14564 Athens, Greece; 6 Laiko General Hospital, 1st Department of Medicine, School of Medicine, National and Kapodistrian University of Athens, 15127 Athens, Greece; katergouf@yahoo.gr; 7 Evangelismos General Hospital 1st Department of Intensive Care Medicine, National & Kapodistrian University of Athens , 10676 Athens, Greeceannkyr@med.uoa.gr (A.K.); 8 University Hospital of Heraklion, Internal Medicine Department, 71110 Heraklion, Greece; skarakonstantis@uoc.gr; 9 Korgialenio-Mpenakio General Hospital, Intensive Care Unit, 11526 Athens, Greece; 10 Chest Diseases Hospital ‘I Sotiria’, Intensive Care Unit, 11527 Athens, Greece; 11 University Hospital of Ioannina, Intensive Care Unit, 45500 Ioannina, Greece; 12 Hippokration General Hospital, 2nd Department of Internal Medicine, School of Medicine, National & Kapodistrian University of Athens, 11527 Athens, Greece; 13 Aghios Andreas Hospital, Intensive Care Unit, 26336 Patra, Greece; 14 Laiko General Hospital, Intensive Care Unit, 11527 Athens, Greece; 15 National and Kapodistrian University of Athens, 11527 Athens, Greece; 16 University Hospital of Heraklion, Intensive Care Unit, University of Crete, 71500 Heraklion, Greece

**Keywords:** *Acinetobacter baumannii*, pandrug resistance, carbapenem resistance, pneumonia, bloodstream infection, sulbactam, tigecycline, colistin, mortality, clinical failure

## Abstract

**Background:** Pandrug-resistant (PDR) *Acinetobacter baumannii* represents a major therapeutic challenge in regions where carbapenem-resistant *A. baumannii* (CRAB) is endemic. Whether the PDR phenotype independently worsens clinical outcomes beyond the effects of disease severity and therapeutic limitations remains uncertain. This study compared the characteristics, management, and outcomes of severe infections caused by PDR and carbapenem-resistant, colistin-susceptible *A. baumannii*. **Methods:** We conducted a retrospective analysis of prospectively collected data across 11 tertiary-care hospitals in Greece (February 2022–June 2024). Consecutive adults with bloodstream infection or hospital-acquired/ventilator-associated pneumonia caused by CRAB or PDR *A. baumannii* were enrolled. The primary outcome was 14-day clinical failure; secondary outcomes included 28-day mortality, microbiological eradication, organ dysfunction, and organ-support-free days. Multivariable logistic and Cox regression analyses, before and after propensity score matching, were performed to adjust for confounding. **Results:** Among 142 patients, 91 (64%) had PDR and 51 (36%) had carbapenem-resistant, colistin-susceptible infections. Clinical failure occurred in 41% of patients and did not differ significantly between PDR and CRAB infections (39% vs. 45%; *p* = 0.440). Twenty-eight-day mortality was 33% and 22%, respectively (*p* = 0.139). After adjustment, the PDR phenotype was not independently associated with clinical failure or mortality. Higher APACHE II score and pneumonia independently predicted clinical failure, whereas sulbactam-containing therapy was associated with lower odds of failure (OR 0.24, 95% CI 0.07–0.79). Older age, higher SOFA score, impaired lactate clearance, and tigecycline-containing therapy independently predicted 28-day mortality. In matched analysis, PDR showed a non-significant upward trend in 28-day mortality (HR 2.36, 95% CI 0.97–5.76; *p* = 0.059). **Conclusions:** In severe *A. baumannii* infections, the PDR phenotype was not an independent determinant of clinical failure or short-term mortality. Patient severity and antimicrobial strategy were major outcome correlates; treatment associations should be interpreted cautiously given the observational design.

## 1. Introduction

*Acinetobacter baumannii* has emerged as one of the most challenging healthcare-associated pathogens worldwide and is a leading cause of hospital-acquired pneumonia (HAP), including ventilator-associated pneumonia (VAP), ventilated hospital-acquired pneumonia (vHAP), bloodstream infection (BSI), and other severe infections among critically ill patients. Its remarkable ability to survive in the hospital environment, acquire multiple antimicrobial resistance determinants, and persist under selective antimicrobial pressure has facilitated its global dissemination, particularly in intensive care units (ICUs) [1,2]. Over the past two decades, carbapenem-resistant *A. baumannii* (CRAB), predominantly mediated by OXA-type carbapenemases [3], has become endemic in many regions, especially in Southern Europe, where Greece consistently reports among the highest resistance rates worldwide [4]. Consequently, the World Health Organization has designated CRAB as a critical-priority pathogen, highlighting the need for the development of new antimicrobial therapies [5].

Severe CRAB infections are associated with prolonged hospitalization, increased healthcare costs, and substantial mortality, particularly in mechanically ventilated and critically ill patients [6,7]. Mortality in patients with CRAB infection is influenced not only by antimicrobial resistance. Host-related factors including baseline illness severity, septic shock, underlying comorbidities, and the appropriateness of empirical antimicrobial therapy are major determinants of prognosis [8].

An additional challenge is the emergence of pandrug-resistant (PDR) *A. baumannii*, defined as non-susceptibility to all routinely available antimicrobial classes, including polymyxins and tigecycline [9,10]. In countries where CRAB is endemic, PDR isolates are increasingly encountered as a consequence of sustained antimicrobial pressure and prolonged healthcare exposure. During the study period in Greece, access to newer therapeutic agents such as cefiderocol and sulbactam-durlobactam was limited, leaving clinicians largely dependent on combinations of older antimicrobial agents with uncertain clinical efficacy [11,12].

Despite the obvious therapeutic implications of pandrug resistance, its independent prognostic significance remains unclear. PDR infection is generally perceived as representing a more severe clinical entity because treatment options are extremely limited [10,11]. However, direct comparison of outcomes between PDR and carbapenem-resistant, colistin-susceptible *A. baumannii* infections is limited. Most available reports have evaluated CRAB infections as a particular group or have focused exclusively on PDR isolates, making it difficult to determine whether the PDR phenotype itself contributes to adverse outcomes or simply reflects prolonged hospitalization, cumulative antimicrobial exposure, and greater patient complexity [13,14,15].

Traditionally, colistin has been the cornerstone of therapy for CRAB infection, but its clinical utility is constrained by nephrotoxicity, limited pharmacodynamic exposure, uncertain efficacy in lung infections, and increasing resistance [16,17]. During the study period, international treatment recommendations increasingly favored sulbactam-based regimens for CRAB infections because sulbactam possesses intrinsic antibacterial activity against *A. baumannii* through high-affinity binding to penicillin-binding proteins 1 and 3 [18]. In the absence of sulbactam-durlobactam, current international guidelines recommend high-dose ampicillin-sulbactam combined with one or more additional agents for the treatment of severe CRAB infections [18,19]. Nevertheless, comparative evidence regarding the effectiveness of different combination regimens remains limited, particularly in settings where therapeutic options are restricted to conventional antimicrobials [18,19].

We therefore conducted a retrospective analysis of prospectively collected data within the ‘DEaling with Severe Problematic drug-resistant Acinetobacter Infections Regionally’ (DESPAIR) project, which stands as a multicentre observational cohort study across tertiary-care hospitals in Greece, to compare the clinical characteristics, antimicrobial management, and outcomes of severe bloodstream infections and HAP/VAP caused by carbapenem-resistant, colistin-susceptible or PDR *A. baumannii*. We hypothesized that, after adjustment for differences in patient characteristics, illness severity, infection syndrome, and antimicrobial treatment, the PDR phenotype would not be independently associated with clinical failure or mortality. In addition, we evaluated microbiological response, longitudinal changes in organ dysfunction, organ-support-free days, and the association between commonly used antimicrobial regimens and clinical outcomes in a high-endemic setting where treatment options were largely limited to conventional agents.

## 2. Materials and Methods

### 2.1. Study Design and Setting

We conducted a retrospective analysis of prospectively collected data in 12 clinical departments across 11 tertiary-care hospitals in Greece between February 2022 and June 2024 within the DESPAIR study network. A previous analysis of DESPAIR participants with PDR infection, focusing on the comparative effectiveness of two targeted antimicrobial regimens, has been reported [20]. The study was coordinated by the Hellenic Study Group for the Combat of Antimicrobial Resistance in Critically Ill Patients under the auspices of the Hellenic Society of Chemotherapy. The primary objective was to compare the clinical characteristics, antimicrobial management, and outcomes of severe infections caused by carbapenem-resistant, colistin-susceptible (CRAB) and PDR *A. baumannii*.

### 2.2. Study Population

Consecutive adult patients (≥18 years) admitted to either intensive care units (ICUs) or general wards were eligible if they had a first episode of monomicrobial BSI, HAP, VAP, or vHAP caused by carbapenem-resistant *A. baumannii*.

Bloodstream infection was defined by the isolation of *A. baumannii* from at least one blood culture in the presence of compatible clinical findings. Pneumonia was diagnosed according to clinical and radiological criteria together with quantitative bronchial secretions culture meeting established diagnostic thresholds [21]. Only the first eligible infection episode per patient was included. Patients receiving concomitant systemic antimicrobial therapy directed exclusively against Gram-positive pathogens or adjunctive nebulized antimicrobial therapy were excluded.

### 2.3. Microbiological Methods

Species identification and antimicrobial susceptibility testing were performed in the microbiology laboratories of participating hospitals using locally validated routine methods. Sites were encouraged to perform colistin susceptibility testing by broth microdilution, in accordance with the recommendations of the European Committee on Antimicrobial Susceptibility Testing (EUCAST; version 14.0). Patients were classified according to the antimicrobial susceptibility profile of the index isolate. Local laboratory protocols required testing of carbapenems and colistin but did not mandate systematic susceptibility testing of all other antimicrobial agents with potential in vitro activity against *A. baumannii*. CRAB was defined as carbapenem-resistant and colistin-susceptible *A. baumannii*. PDR *A. baumannii* was defined pragmatically and operationally as an isolate that was non-susceptible to both carbapenems and colistin and was reported resistant to all locally tested antimicrobials, which included tigecycline, ampicillin-sulbactam and an aminoglycoside. Because cefiderocol and sulbactam-durlobactam were not available in Greece during the study period, susceptibility to these agents was not routinely determined. Thresholds for non-susceptibility were set at MIC > 8/4 mg/L and MIC > 2 mg/L for ampicillin-sulbactam and tigecycline, respectively. The former was in accordance with CLSI guidance for colistin (CLSI M100, 34th Ed., 2024), while the latter was a study-defined operational threshold, consistent with the DESPAIR protocol and the previous DESPAIR analysis [20].

### 2.4. Data Collection

Clinical data were prospectively collected using standardized electronic case report forms developed in REDCap (Research Electronic Data Capture) and hosted by the National and Kapodistrian University of Athens. Baseline variables included demographic characteristics, comorbidities, Charlson Comorbidity Index, McCabe–Jackson classification, admission related to coronavirus disease 2019 (COVID-19), hospital location (ICU or ward), illness severity (Acute Physiology and Chronic Health Evaluation II [APACHE II] score at admission and Sequential Organ Failure Assessment [SOFA] score at infection onset), laboratory findings, organ-support requirements, microbiological characteristics, and antimicrobial treatment. Treatment variables included empirical and targeted antimicrobial therapy, number of administered agents, microbiological activity of empirical treatment, duration of therapy, and the antimicrobial backbone of targeted combination regimens.

### 2.5. Definitions

Healthcare-associated infections were defined according to the Centers for Disease Control and Prevention/National Healthcare Safety Network (CDC/NHSN) surveillance definitions [22]. Sepsis and septic shock were defined according to the Third International Consensus Definitions for Sepsis and Septic Shock (Sepsis-3) [23]. The underlying illnesses were classified as rapidly fatal, ultimately fatal, and nonfatal according to the modified McCabe and Jackson classification [24]. Infection onset was defined as the date of collection of the index positive culture. Empirical antimicrobial therapy referred to antimicrobial treatment initiated before antimicrobial susceptibility results became available, whereas targeted therapy referred to treatment administered after susceptibility testing had been reported.

Clinical failure at Day 14 was defined by the occurrence of one or more of the following: (i) all-cause mortality before Day 14; (ii) persistent bacteremia beyond Day 5 among patients with bloodstream infection; (iii) change in antimicrobial therapy because of clinical deterioration; (iv) discontinuation of one or more antimicrobial agents because of treatment-related toxicity; or (v) absence of improvement in oxygenation by Day 14 among patients with pneumonia.

Microbiological eradication was defined as a negative follow-up culture obtained between days 6 and 14 or presumed eradication when repeat cultures were not clinically indicated. Recurrent infection was defined as recurrence of *A. baumannii* infection within 28 days from infection onset. Superinfection was defined as infection caused by a different pathogen during follow-up.

### 2.6. Outcomes

The primary outcome was clinical failure by Day 14. Secondary outcomes included all-cause 28-day mortality, microbiological eradication among microbiologically evaluable patients, recurrent infection, superinfection, longitudinal changes in SOFA score, and organ-support-free days through Day 28.

Among patients admitted to the ICU, ICU-free, ventilator-free, vasopressor-free, continuous renal replacement therapy (CRRT)-free, and overall organ-support-free days were calculated through Day 28.

### 2.7. Statistical Analysis

The collected data were subjected to descriptive analyses. Continuous variables are presented with mean values and standard deviations or medians and interquartile ranges, and compared with either parametric or non-parametric tests, as appropriate. Categorical variables are summarized with counts and proportions, and between-group comparisons were done with χ2 or Fisher’s exact test.

Univariate and multivariate logistic regression analyses were employed to evaluate factors associated with the primary outcome, and the respective odds ratios (ORs) with 95% confidence intervals (CI) were calculated. Variable selection for the multivariable model was performed based on their statistical association with the primary outcome and their clinical relevance. As per our aim, the resistance phenotype was forced into the model. The goodness-of-fit of the candidate models was assessed with the Akaike Information Criterion.

The robustness of the analyses was evaluated with two post hoc sensitivity analyses. To account for uncertainty posed by suboptimal testing for colistin susceptibility in a minority of patients, only cases in whom broth microdilution was employed were subjected to multivariate modeling. Furthermore, since ampicillin-sulbactam and tigecycline-containing regimens were coded as non-mutually exclusive binary variables, to mitigate ambiguity generated by combination treatment (i.e., patients receiving both agents), a second post hoc sensitivity analysis was performed, including patients not concurrently receiving these agents as targeted treatment.

To account for baseline imbalances, propensity matching was performed within an additional analysis. Specifically, the probability of a patient infected by a PDR strain was modeled as a function of disposition within the hospital (ICU vs. ward), presence of sepsis/septic shock, SOFA score at infection onset, infection type (HAP vs. vHAP vs. VAP vs. BSI), the McCabe-Jackson classification and APACHE II score on ICU/ward admission and calculated for each patient. One-to-one nearest-neighbor matching, without replacement, was performed with a caliper of 0.05 of the raw probability. Thirty-eight matched pairs were produced. Standardized mean differences were calculated both before and after the propensity matching. Clinically relevant variables with post-matching SMDs ≥ 0.2 were considered for introduction into the subsequent models as covariates, to reduce bias.

Unadjusted survival analyses were performed with the Kaplan–Meier method and between-group differences were compared with the log-rank test. Univariable and multivariable Cox regression analyses were employed, and hazard ratios (with 95% CΙs) were calculated. Variable selection followed the same principles as for the primary outcome. Adjusted survival analyses were also performed in the propensity-matched population.

The propensity matching procedure mitigated baseline imbalances, as evidenced by a reduction in standardized mean differences in key variables, such as patient disposition and infection type (See ‘Matching procedure and matched cohorts’ section in Supplement). To account for the persistent imbalance in admission APACHE II score and age, these variables were forced into the adjusted logistic regression model, while in the adjusted Cox regression model only APACHE II was introduced due to the lower number of events (n = 22, allowing for a total of two variables to avoid overfitting).

The trajectory of organ dysfunction per resistance phenotype was compared using a linear mixed model where SOFA score (at infection onset and days 7 and 14) was the dependent variable and resistance phenotype, time and their interaction were entered as covariates. Resource utilization indices were calculated for patients in the ICU at infection onset by summing the days each modality (ICU hospitalization, ventilator, vasopressors, continuous renal replacement therapy) was used up to day twenty-eight. To adjust for death being a competing event, the value of −1 was assigned to intervention-free days in non-survivors [25].

Analyses were performed using available cases, without imputation of missing values. The denominators used for each variable, subgroup outcome, and regression analysis were reported when they differed from the total cohort size. No formal a priori sample-size calculation was undertaken, as the study included all consecutive eligible patients enrolled during the predefined study period. The intention-to-treat principle was followed, and analyses were performed with IBM SPSS Statistics (v26.0) and RStudio version 2026.05.1 Build 225. RStudio’s *MatchIt* library was used for the matching procedure.

### 2.8. Ethics

The study was approved by the Institutional Review Board or Ethics Committee of each participating center and was conducted in accordance with the ethical principles of the Declaration of Helsinki. Because of the observational nature of the study, the requirement for written informed consent was waived in accordance with national regulations and local institutional policies.

## 3. Results

### 3.1. Patient Characteristics

A total of 142 patients were included in the study, of whom 91 (64%) had PDR *A. baumannii* infection and 51 (36%) had carbapenem-resistant, colistin-susceptible *A. baumanni* infection. The median age was 69 years (IQR, 59.8–77.0), and 83 patients (59%) were male. All 142 patients were evaluable for day-14 clinical failure. Day-28 vital-status follow-up was available for 141 patients (51 CRAB and 90 PDR); one patient in the PDR group had unavailable day-28 vital status. Baseline demographic characteristics, comorbidity burden, Charlson Comorbidity Index, McCabe–Jackson classification, and admission APACHE II scores were comparable between the two groups (Table 1).

Overall, 129 patients (91%) were admitted to ICU. Although illness severity at infection onset was similar, with a median SOFA score of 8 in both groups, patients with CRAB infection were more frequently managed in the ICU (100% vs. 86%; *p* = 0.005), required invasive mechanical ventilation (96% vs. 74%; *p* = 0.001), and received vasopressor support (80% vs. 59%; *p* = 0.011) than patients with PDR infection (Table 1).

Pneumonia was the predominant infection syndrome, accounting for 102 cases (72%), whereas 40 patients (28%) had BSI. The distribution of infection syndromes was similar between groups, with pneumonia occurring in 67% of patients with CRAB and 75% of patients with PDR infections. Among patients with pneumonia, VAP accounted for 79% of episodes, followed by vHAP (12%) and non-ventilated HAP (9%). Bacteremic pneumonia occurred in 55% of pneumonia cases. At infection onset, 66 patients (47%) had sepsis and 58 (41%) septic shock. Infection characteristics are depicted in Table 1.

All 51 isolates in the CRAB group were susceptible to colistin. Susceptibility rates to ampicillin-sulbactam, tigecycline and amikacin were 4.8% (1/21), 7% (3/43) and 0% (0/47), respectively. All 91 isolates in the PDR group were non-susceptible to colistin. Similarly, all isolates with available susceptibility testing were non-susceptible to ampicillin-sulbactam (46 isolates), tigecycline (86 isolates), and amikacin (88 isolates).

Colistin susceptibility was determined by broth microdilution in 116/131 isolates (88.5%; in 11 cases, the method was not reported), including 36/46 CRAB isolates (78.3%) and 80/85 PDR isolates (94.1%). The remaining 15 isolates were tested using locally validated automated susceptibility systems (n = 13) or gradient-diffusion methods (n = 2). Because non-reference colistin susceptibility testing may introduce phenotype misclassification, a post hoc sensitivity analysis restricted to isolates evaluated by broth microdilution was performed.

### 3.2. Antimicrobial Therapy

Empirical antimicrobial therapy was administered to 121 patients; 42 with CRAB and 79 with PDR infection (Table S1). Among the 42 patients with CRAB infection, 16 (38%) received at least one agent with in vitro activity and 26 received no active agent, whereas none of the 79 patients with PDR infection received active empirical therapy.

Targeted antimicrobial therapy also differed between groups (Tables S2 and S3). Nearly all patients (99%, 141/142) received a colistin-containing regimen. Sulbactam-containing therapy was administered to 113 patients (80%), meropenem-containing therapy to 78 (55%), and tigecycline-containing therapy to 36 (25%). The most frequently prescribed targeted regimen was colistin plus ampicillin-sulbactam plus meropenem (46%), predominantly among patients with PDR infection, whereas dual therapy with colistin plus ampicillin-sulbactam was more commonly used in patients with CRAB infection.

### 3.3. Clinical Outcomes

**Figure 1:**
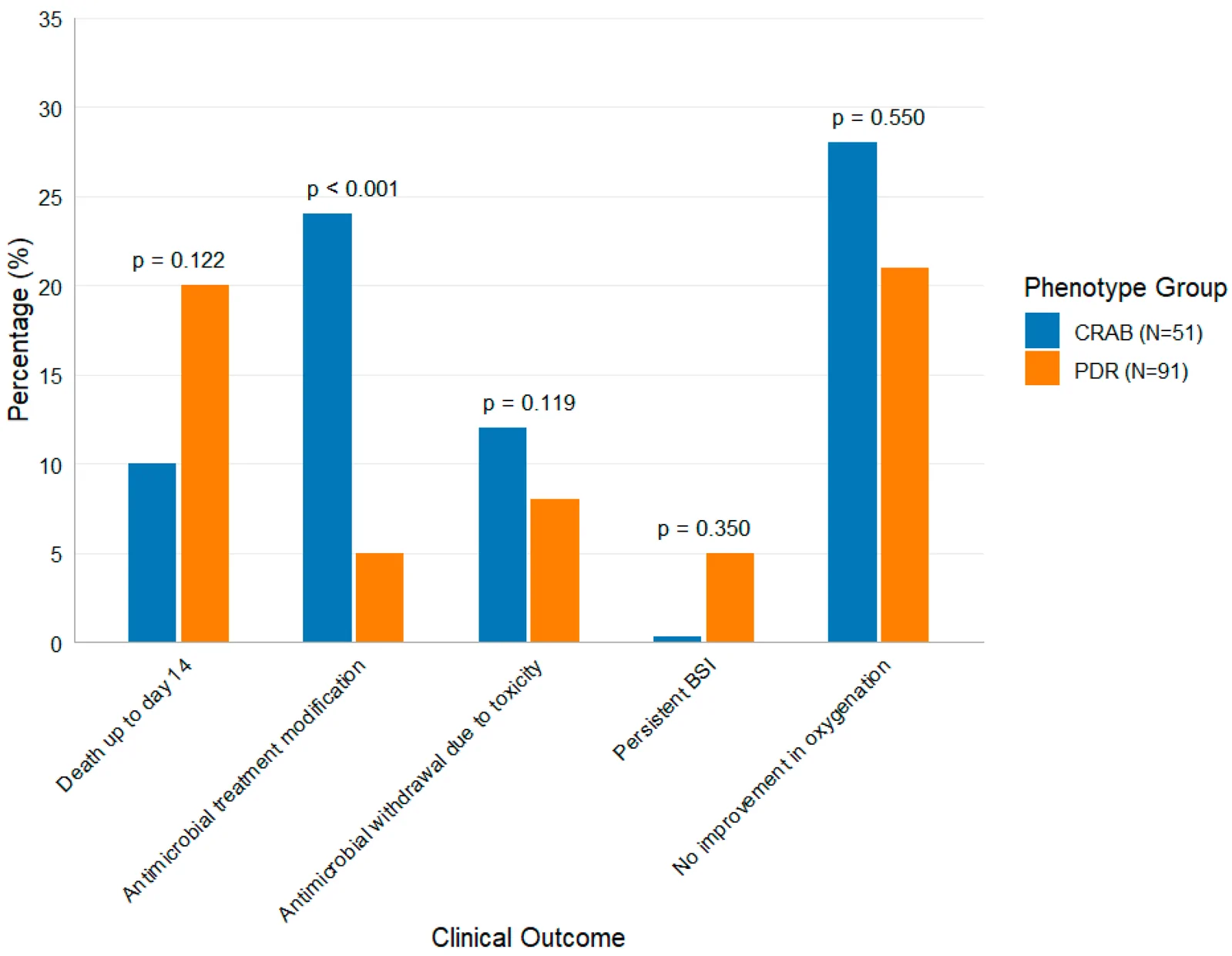
Reasons for clinical failure on day 14 among 142 patients with *Acinetobacter baumannii* infection according to resistance phenotype. Clinical failure at Day 14 was defined by the occurrence of one or more of the following: (i) all-cause mortality up to day 14; (ii) persistent bacteremia beyond day 5 among patients with bloodstream infection; (iii) modification of antimicrobial therapy due to clinical deterioration; (iv) discontinuation of one or more antimicrobial agents because of treatment-related toxicity; or (v) absence of improvement in oxygenation by Day 14 among patients with pneumonia. Reasons for failure are shown as one per patient. Reasons for clinical failure were not mutually exclusive; therefore, an individual patient could contribute to more than one failure category, for example, modification of antimicrobial treatment and persistent bacteremia. BSI, Bloodstream infection; CRAB, Carbapenem-resistant *Acinetobacter baumannii*; PDR, Pandrug-resistant.

Clinical failure by Day 14 occurred in 41% of patients (58 out of 142), and there was no significant difference between patients with CRAB or PDR infection (45% and 39%, respectively; *p* = 0.440) (Table 2). Analysis of the individual components of clinical failure demonstrated some differences between groups; 14-day mortality was numerically higher among patients with PDR infection than among those with CRAB infection (20% vs. 10%), although the difference did not reach statistical significance (*p* = 0.122). Also, antimicrobial therapy was modified, due to clinical deterioration, more frequently in patients with CRAB infection than in those with PDR (24% vs. 5%; *p* < 0.001). No other significant differences were observed between resistance phenotypes; treatment discontinuation because of drug-related toxicity occurred in 9% of patients, persistent bacteremia was documented in only one patient, while lack of improvement in oxygenation occurred in 24% of evaluable patients with pneumonia (Table 2; Figure 1).

**Figure 2:**
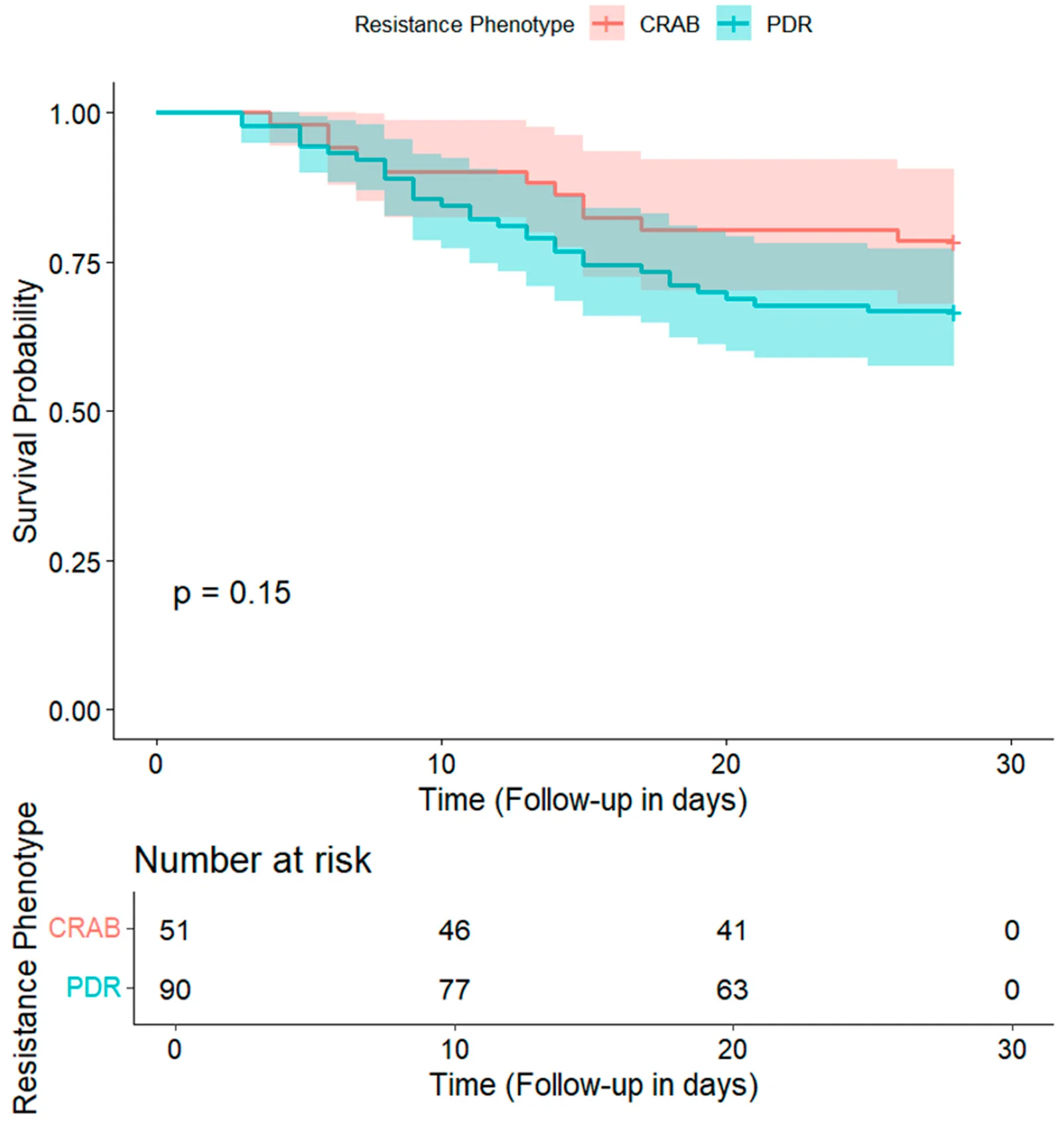
Survival curves of 141 patients with *Acinetobacter baumannii* infection according to their resistance profile. CRAB: Carbapenem-resistant *Acinetobacter baumannii*; PDR: Pandrug-resistant.

Among the 141 patients evaluable for survival, 28-day all-cause mortality was 29%. Mortality was numerically higher in the PDR group than in the CRAB group (33% vs. 22%), but this difference was not statistically significant (*p* = 0.139). Likewise, Kaplan–Meier survival analysis demonstrated no significant difference in 28-day survival between the two groups (log-rank *p* = 0.150) (Figure 2).

### 3.4. Microbiological Response and Organ-Support Outcomes

Among the 127 patients who were microbiologically evaluable, eradication was achieved in 104 (82%). Eradication was more frequent among patients with a BSI than among those with pneumonia (97% versus 75%), and was higher in the CRAB group than in the PDR group (89% versus 78%) (Table S4). However, neither of these comparisons was statistically significant. Recurrent infection and superinfection occurred in 6% and 12% of patients, respectively, with no significant differences according to resistance phenotype (Table 2).

Among survivors, organ dysfunction improved progressively during follow-up. Mean SOFA score decreased from 8.13 at infection onset to 6.83 on day 7 and 5.52 on Day 14, corresponding to an overall reduction of 2.61 points. The magnitude of improvement was similar in the CRAB and PDR groups (reduction of 2.57 and 2.66 SOFA points, respectively) (Table S5, igure S1). Similarly, ICU-free, ventilator-free, vasopressor-free, CRRT-free, and overall organ-support-free days did not differ significantly between the two groups (Table S4).

### 3.5. Predictors of 14-Day Clinical Failure

In univariable logistic regression analysis, higher APACHE II score at admission, pneumonia, CRRT, higher serum creatinine, higher SOFA score at infection onset, and no administration of sulbactam-containing therapy were associated with clinical failure by Day 14, whereas resistance phenotype had no association with clinical outcome (Table 3). In the multivariable model, independent predictors of clinical failure were: higher APACHE II score at admission (OR per one-point increase, 1.10; 95% CI, 1.04–1.18; *p* = 0.003) and pneumonia (OR 3.86; 95% CI, 1.37–12.65; *p* = 0.016). Sulbactam-containing therapy was independently associated with lower odds of clinical failure (OR 0.24; 95% CI, 0.07–0.79; *p* = 0.023). Resistance phenotype was not a predictor of clinical failure (OR 1.15, 95% CI, 0.40–3.44; *p* = 0.800). The final multivariable model for 14-day clinical failure is shown in Table 4. In the post hoc sensitivity analysis restricted to patients whose isolates were tested for colistin susceptibility by broth microdilution, the PDR phenotype remained unassociated with day-14 clinical failure (OR 0.68; 95% CI, 0.18–2.65; *p* = 0.567) (Table S6). In a separate post hoc sensitivity analysis excluding patients who concurrently received ampicillin-sulbactam and tigecycline, the association between sulbactam-containing therapy and lower odds of clinical failure remained significant (OR 0.24; 95% CI, 0.06–0.88; *p* = 0.032) (Table S7).

### 3.6. Predictors of 28-Day Mortality

The results of univariate Cox proportional hazards regression analysis appear in Table S8. In the multivariable model, older age (HR per year, 1.04; 95% CI, 1.01–1.07; *p* = 0.010), higher SOFA score at infection onset (HR per point, 1.25; 95% CI, 1.13–1.38; *p* < 0.001), and impaired lactate kinetics (HR 1.32; 95% CI, 1.02–1.70; *p* = 0.032) were associated with mortality. Tigecycline-containing therapy was independently associated with a higher hazard of 28-day mortality (HR 2.09; 95% CI, 1.01–4.32; *p* = 0.046) (Table 5). The PDR phenotype showed a non-significant upward trend in 28-day mortality (HR 2.03; 95% CI, 0.94–4.39; *p* = 0.073). These results proved essentially robust in the two post hoc sensitivity analyses (Tables S9 and S10).

### 3.7. Propensity Score-Matched Analysis

Propensity score matching yielded 38 balanced pairs of patients with CRAB and PDR infection. The resistance phenotype was not associated with clinical failure by day 14 (OR 0.69; 95% CI, 0.26–1.82; *p* = 0.451). In the matched Cox analysis, PDR infection showed a non-significant upward trend in 28-day mortality (HR 2.36; 95% CI, 0.97–5.76; *p* = 0.059). Detailed results of the propensity score-matched analyses are presented in the Supplementary Material (Tables S11–S15 and Figure S2).

## 4. Discussion

In this retrospective analysis of prospectively collected data from a multicentre cohort with severe *A. baumannii* infections from a country where carbapenem resistance is endemic, we found that the PDR phenotype was not independently associated with either 14-day clinical failure or 28-day mortality after adjustment for illness severity, infection syndrome, and treatment-related factors. In the propensity score-matched analysis, PDR remained unassociated with clinical failure, but showed a non-significant upward trend in mortality. Instead, prognosis was determined predominantly by host-related factors, including illness severity and organ dysfunction, whereas sulbactam-containing therapy was independently associated with lower odds of clinical failure.

These findings address an important knowledge gap regarding the clinical significance of pandrug resistance. Because therapeutic options for PDR *A. baumannii* are extremely limited [10,11], these infections are often perceived as representing a more severe biological entity than carbapenem-resistant, colistin-susceptible isolates. However, evidence supporting this assumption has remained limited. Our results suggest that progressive antimicrobial resistance primarily complicates antimicrobial management rather than independently worsens prognosis, once the severity of the host response and infection characteristics are taken into account. This interpretation is consistent with contemporary observational studies demonstrating that mortality among patients with CRAB infection is driven predominantly by acute illness severity, organ dysfunction, septic shock, and adequacy of antimicrobial therapy rather than by resistance phenotype alone [26,27].

The biological explanation for these observations is likely multifactorial [28,29,30,31,32,33]. Progressive acquisition of antimicrobial resistance does not necessarily confer increased bacterial virulence [29,30,33]. Rather, the transition from CRAB to PDR probably reflects sequential acquisition of additional resistance determinants within successful hospital-adapted clones under sustained antimicrobial pressure, resulting in progressive loss of therapeutic options without a corresponding increase in pathogenic potential [31]. This concept is supported by findings from an exploratory sub-group analysis of the AIDA study, in which colistin-resistant CRAB isolates were not associated with increased mortality after adjustment for confounding factors [28]. Whole-genome sequencing performed within the AIDA program further demonstrated that CRAB infections in Mediterranean countries, including Greece, were predominantly caused by internationally disseminated sequence type 2 (ST2) clones, indicating that additional resistance mechanisms accumulate within established epidemic lineages rather than generating novel hypervirulent strains [29]. Similarly, comparative genomic analyses have shown that virulence-associated gene profiles remain largely conserved despite differences in antimicrobial susceptibility [30]. Finally, the study by Karakonstantis and colleagues demonstrated that PDR isolates frequently coexist or evolve from CRAB isolates within the same patient, supporting the concept that pandrug resistance represents a dynamic adaptive phenotype rather than a distinct biological entity with enhanced virulence [31]. Collectively, these observations provide a plausible biological explanation for our findings and support the concept that antimicrobial resistance and bacterial virulence evolve largely independently.

Pneumonia emerged as an independent predictor of clinical failure, highlighting the particular therapeutic challenges associated with pulmonary *A. baumannii* infections. More than two-thirds of patients in our cohort had HAP or VAP, reflecting the epidemiology of *A. baumannii* infection in critically ill patients. High bacterial inocula, impaired pulmonary host defenses, biofilm formation on endotracheal tubes, and the limited epithelial lining fluid penetration of several conventional antimicrobial agents probably contribute to the reduced effectiveness of treatment in this setting [34,35,36,37]. The predominance of VAP in our cohort likely further contributed to these observations. Our findings are consistent with the post hoc analysis of the OVERCOME trial, which demonstrated poorer outcomes among patients with pneumonia caused by isolates exhibiting higher colistin minimum inhibitory concentrations (MIC) during colistin monotherapy, emphasizing the limitations of polymyxin-based treatment for severe pulmonary infections [38].

Another important finding of the present study was the independent association between sulbactam-containing therapy and reduced risk of clinical failure [39]. Unlike other β-lactamase inhibitors, sulbactam possesses intrinsic antibacterial activity against *A. baumannii* through high-affinity binding to penicillin-binding proteins 1 and 3, providing a strong biological rationale for its continued therapeutic role despite widespread multidrug resistance [18,19]. During the study period, sulbactam-durlobactam and cefiderocol were not routinely available in Greece; consequently, high-dose ampicillin-sulbactam represented the cornerstone of combination therapy. Although our observational design precludes causal inference regarding comparative treatment efficacy, the observed association between sulbactam-containing regimens and lower clinical-failure risk is consistent with current international recommendations, which prioritize sulbactam-based combinations for severe CRAB infections when newer agents are unavailable [18,19]. More recently, the ATTACK trial demonstrated superior clinical outcomes with sulbactam-durlobactam compared with colistin-based therapy, further reinforcing the significant role of sulbactam-based treatment strategies in the management of CRAB infections [40].

Conversely, tigecycline-containing therapy was independently associated with increased 28-day mortality. This finding should be interpreted cautiously because treatment allocation was not randomized and residual confounding by indication cannot be excluded; clinicians may have preferentially selected tigecycline for patients with the most limited therapeutic alternatives. Nevertheless, the association persisted after multivariable adjustment and is biologically plausible. Tigecycline achieves relatively low serum concentrations and has recognized pharmacokinetic limitations in bacteremia, severe pneumonia, and other high-inoculum infections [41,42]. Similar findings have been reported in recent multicentre observational studies [39,43], including a large Italian cohort of patients with CRAB bloodstream infection in which tigecycline-containing regimens were associated with higher mortality whereas cefiderocol-containing therapy was associated with improved survival [43]. Together with our previous DESPAIR analysis demonstrating superior outcomes with combinations containing colistin, high-dose ampicillin-sulbactam, and meropenem compared with tigecycline-based regimens [20], these findings support current guideline recommendations that discourage tigecycline as the principal component of therapy for severe CRAB infections whenever more effective alternatives are available.

Our study has several important strengths. To our knowledge, this is the first multicentre analysis of prospectively collected cohort data specifically designed to compare clinical outcomes between PDR and carbapenem-resistant, colistin-susceptible *A. baumannii* infections using standardized definitions and prospectively collected clinical data. The inclusion of patients from multiple tertiary-care hospitals enhances the external validity of our findings in regions where CRAB is endemic. Furthermore, the evaluation of multiple clinically relevant outcomes, including clinical failure, microbiological eradication, longitudinal organ dysfunction, and organ-support-free days, provides a comprehensive assessment of disease burden beyond mortality alone.

Several limitations should also be acknowledged. First, the observational design precludes definitive conclusions regarding treatment efficacy, and residual confounding cannot be completely excluded despite multivariable adjustment and propensity score-matched sensitivity analyses. Second, antimicrobial dosing, therapeutic drug monitoring, timing of antimicrobial administration, and source-control interventions were not standardized across participating centers. Third, molecular characterization of resistance mechanisms and bacterial fitness or virulence determinants was not systematically performed, limiting mechanistic interpretation of our findings. Fourth, gold-standard broth microdilution for colistin susceptibility was not performed in all infections, raising the possibility that some PDR infections ‘invaded’ the CRAB population. Nevertheless, broth microdilution adoption was high, and a post hoc sensitivity analysis including only these patients did not refute the findings. Fifth, treatment components were overlapping because combination regimens were common. However, in a sensitivity analysis in patients in whom ampicillin-sulbactam and tigecycline were not combined as targeted treatment, the results remained essentially unaltered. Sixth, no a priori sample-size calculation was performed, and the sample size and number of outcome events limited the precision of adjusted and matched analyses, particularly for mortality. Seventh, the absence of centralized microbiological testing precluded uniform and exhaustive susceptibility testing across all isolates. Consequently, some isolates classified as PDR may have been misclassified because antimicrobial panels differed between participating laboratories and susceptibility to all potentially active agents was not systematically assessed. Nevertheless, this limitation also reflects the real-world nature of the study, in which therapeutic decisions were based on the resistance phenotype reported by each local microbiology laboratory at the time of clinical care. Thus, the PDR classification used in this cohort should be interpreted as a pragmatic, clinically applied phenotype rather than as confirmation of pandrug resistance against a fully standardized antimicrobial panel. Finally, because cefiderocol and sulbactam-durlobactam were not routinely available during the study period, our results primarily reflect treatment strategies based on conventional antimicrobial combinations and should therefore be interpreted within this therapeutic context.

In conclusion, the PDR phenotype was not independently associated with clinical failure or short-term mortality among patients with severe *A. baumannii* infection after adjustment for illness severity and treatment-related factors. Instead, illness severity, organ dysfunction, infection syndrome, and antimicrobial strategy were the principal correlates of outcomes. Sulbactam-containing therapy was associated with lower odds of clinical failure, whereas tigecycline-containing therapy was associated with higher mortality. However, given the observational, non-randomized nature of treatment allocation, these associations should not be interpreted as evidence of comparative efficacy, benefit, or harm. Larger studies incorporating standardized reference susceptibility testing and contemporary antimicrobial options are needed to better define the independent prognostic significance of the PDR phenotype.

## Figures and Tables

**Table 1:** Demographics, history, index infection, and therapy characteristics according to resistance phenotype.

Variable		Total	CRAB (n = 51)	PDR (n = 91)	*p*-Value	SMD
Age, median (IQR)		69.0 (59.8–77.0)	66.0 (60.0–76.0)	69.0 (59.0–77.0)	0.544	0.030
Male gender, n (%)		83 (59)	29 (57)	54 (59)	0.774	0.050
**In ICU at enrollment, n (%)**		**129 (91)**	**51 (100)**	**78 (86)**	**0.005**	**0.577**
COVID-19 admission, n (%)		18 (13)	4 (8)	14 (15)	0.195	0.237
Immunosuppression, n (%)		18 (13)	8 (16)	10 (11)	0.420	0.139
Diabetes mellitus, n (%)		39 (28)	12 (24)	27 (30)	0.626	0.174
Malignancy, n (%)		28 (20)	11 (22)	17 (19)	0.910	0.075
Moderate-severe CKD, n (%)		16 (11)	5 (10)	11 (12)	0.680	0.073
Congestive heart failure, n (%)		23 (16)	9 (18)	14 (15)	0.726	0.061
Coronary heart disease, n (%)		31 (22)	12 (24)	19 (21)	0.714	0.064
COPD, n (%)		38 (27)	14 (28)	24 (26)	0.889	0.024
McCabe-Jackson classification, n (%) (N = 141)	Non-fatal disease	99 (70)	32 (64)	67 (74)	0.390	0.238
	Ultimately fatal disease	33 (23)	15 (30)	18 (20)		
	Rapidly fatal disease	9 (6)	3 (6)	6 (7)		
Charlson comorbidity index, median (IQR)		5.0 (3.0–6.0)	4.0 (3.0–6.0)	5.0 (3.0–6.0)	0.765	0.069
Admission APACHE II score, mean (SD) (N = 141)		20.5 (8.0)	20.1 (7.8)	20.8 (8.1)	0.591	0.088
Infection type, n (%)	BSI	40 (28)	17 (33)	23 (25)	0.306	0.178
	HAP/VAP	102 (72)	34 (67)	68 (75)		
Time from ICU admission (days, N = 129), median (IQR)		8.0 (4.0–14.0)	9.0 (4.0–13.0)	8.0 (3.0–19.0)	0.807	0.236
Pneumonia type, n (%)	VAP	81 (79)	29 (85)	52 (77)	0.329	0.345
	vHAP	12 (12)	4 (12)	8 (12)		
	HAP	9 (9)	1 (3)	8 (12)		
Bacteremic pneumonia, n (%) (N = 102)		56 (55)	16 (47)	40 (59)	0.260	0.237
**Sepsis, n (%)**		**66 (47)**	**28 (55)**	**38 (42)**	**0.014**	**0.579**
**Septic shock, n (%)**		**58 (41)**	**22 (43)**	**36 (40)**		
**Vasopressor use, n (%)**		**95 (67)**	**41 (80)**	**54 (59)**	**0.011**	**0.471**
**Invasive MV, n (%)**		**116 (82)**	**49 (96)**	**67 (74)**	**0.001**	**0.659**
CRRT, n (%)		19 (13)	7 (14)	12 (13)	0.928	0.016
SOFA score, median (IQR) (N = 137)		8 (5–10)	8 (6–10)	8 (4–10)	0.198	0.168
Lactate (mEq/L), median (IQR) (N = 136)		1.4 (0.9–2.0)	1.4 (0.9–2.0)	1.4 (1.0–1.8)	0.911	0.044
Creatinine (mg/dL), median (IQR)		0.9 (0.7–1.4)	0.8 (0.6–1.4)	1.0 (0.7–1.4)	0.563	0.096
Albumin (g/dL), median (IQR) (N = 123)		2.8 (2.5–3.0)	2.7 (2.5–3.0)	2.8 (2.5–3.1)	0.554	0.139
WBC (×10^3^/μL), median (IQR)		13.2 (9.8–17.6)	13.6 (10.2–19.7)	13.0 (9.2–17.6)	0.454	0.162
Empirical treatment receipt, n (%)		121 (85)	42 (82)	79 (87)	0.473	0.124
Number of empirical antimicrobials per patient (median, IQR)		2.0 (1.0–2.0)	2.0 (1.0–2.0)	2.0 (1.0–2.0)	0.842	0.018
Duration of empiric treatment per antibiotic (days), median (IQR)		3.0 (2.0–3.0)	3.0 (2.0–4.0)	2.0 (2.0–3.0)	0.313	0.073

**Bold values indicate statistically significant differences**. McCabe-Jackson classification follows European Center for Disease Prevention and Control guidance [24]: non-fatal, ultimately fatal, and rapidly fatal disease correspond to expected survival of ≥5 years, 1–5 years, and <1 year, respectively. APACHE II, Acute Physiology and Chronic Health Evaluation II; BSI, Bloodstream infection; CKD, Chronic kidney disease; COPD, Chronic obstructive pulmonary disease; COVID-19, Coronavirus disease 2019; CRAB, Carbapenem-Resistant *Acinetobacter baumannii*; HAP, Hospital-acquired pneumonia; ICU, Intensive care unit; IQR, Interquartile range; MV, Mechanical ventilation; PDR, Pandrug-resistant; SD, Standard deviation; SMD, Standardized mean difference; VAP, Ventilator-associated pneumonia.

**Table 2:** Primary and secondary outcomes.

Variable		Total (N = 142)	CRAB (N = 51)	PDR (N = 91)	*p*-Value
Microbiological outcome					
Positive follow-up blood cultures (days 6–14) (N = 37)		1 (3)	0 (0)	1 (5)	0.350
**Follow-up respiratory cultures in pneumonia patients (N = 102), n (%)**	**Taken**	**71 (70)**	**25 (74)**	**46 (68)**	**0.008**
	**No, presumed eradication**	**18 (18)**	**3 (9)**	**15 (22)**	
	**Not taken due to moribund condition before day 6**	**8 (8)**	**1 (3)**	**7 (10)**	
	**Not taken in error**	**3 (3)**	**3 (9)**	**0 (0)**	
	**Unknown**	**2 (2)**	**2 (6)**	**0 (0)**	
Positive follow-up respiratory cultures (days 6–14), (N = 71), n (%, phenotype of recovered strains of *A. baumannii*)		22 (31), 14 (64, PDR) 8 (36, CRAB)	5 (20), 0 (0, PDR) 5 (100, CRAB)	17 (37), 14 (82, PDR) 3 (18, CRAB)	0.140
Clinical outcome					
Clinical failure on day 14, n (%)		58 (41)	23 (45)	35 (39)	0.440
Elements of clinical failure on day 14					
Death up to day 14, n (%)		23 (16)	5 (10)	18 (20)	0.122
**Antimicrobial treatment modification due to clinical deterioration, n (%)**		**16 (11)**	**12 (24)**	**4 (5)**	**<0.001**
Antimicrobial withdrawal due to toxicity, n (%)		13 (9)	6 (12)	7 (8)	0.119
Persistent BSI, (N = 37), n (%)		1 (3)	0 (0)	1 (5)	0.350
Intubation of initially spontaneously breathing patients (N = 17), n (%)		5 (29)	1 (50)	4 (27)	0.496
No improvement in oxygenation (N = 71), n (%)		17 (24)	8 (28)	9 (21)	0.550
Secondary evaluation of clinical outcome					
Indeterminate outcome, n (%)		18 (13)	8 (16)	10 (11)	0.420
Recurrent infection, n (%)		9 (6)	3 (6)	6 (7)	0.867
Superinfection, n (%)		17 (12)	6 (12)	11 (12)	0.955
Mortality					
All-cause 28-day mortality (N = 141 ^†^), n (%)		41 (29)	11 (22)	30 (33)	0.139

**Bold values indicate statistically significant differences.** ^†^ In one patient, survival status at 28 days was unknown. BSI: Bloodstream infection; CRAB: Carbapenem-resistant *Acinetobacter baumannii*; PDR: Pandrug-resistant.

**Table 3:** Univariable logistic regression analysis of clinical failure by day 14 after infection onset.

Variable		Clinical Failure		Total	OR (95% CI)	*p*-Value
		No (N = 84)	Yes (N = 58)			
Resistance phenotype (PDR vs. CRAB)		56 (67)	35 (60)	91 (64)	0.761 (0.380–1.524)	0.441
Age, median (IQR)		68 (60–75)	72 (62–78)	69 (60–77)	1.017 (0.993–1.042)	0.175
Female gender, n (%)		33 (39)	26 (45)	59 (42)	1.256 (0.637–2.473)	0.510
ICU allocation (vs. ward), n (%)		76 (91)	53 (91)	129 (91)	0.896 (0.278–2.891)	0.855
COVID-19, n (%)		8 (10)	10 (17)	18 (13)	1.979 (0.730–5.366)	0.180
Immunosuppression, n (%)		11 (13)	7 (12)	18 (13)	0.911 (0.331–2.508)	0.857
McCabe-Jackson classification, n (%)	Non- fatal disease	63 (75)	36 (63)	99 (70)	Reference	0.058
	**Ultimately fatal disease**	**14 (17)**	**19 (33)**	**33 (23)**	**2.375 (1.064–5.300)**	**0.035**
	Rapidly fatal disease	7 (8)	2 (4)	9 (6)	0.500 (0.099–2.537)	0.403
Diabetes mellitus, n (%)	None	62 (74)	41 (71)	103 (73)	Reference	0.825
	Uncomplicated	12 (14)	8 (14)	20 (14)	1.008 (0.379–2.680)	0.987
	End-organ damage	10 (12)	9 (16)	19 (13)	1.361 (0.509–3.638)	0.539
Malignancy, n (%)	None	68 (81)	46 (79)	114 (80)	Reference	0.864
	Any leukemia, lymphoma or localized solid tumor	12 (14)	8 (14)	20 (14)	0.986 (0.374–2.599)	0.976
	Metastatic solid tumor	4 (5)	4 (7)	8 (6)	1.478 (0.352–5.211)	0.594
Congestive heart failure, n (%)		16 (19)	7 (12)	23 (16)	0.583 (0.223–1.523)	0.271
COPD, n (%)		19 (23)	19 (33)	38 (27)	1.667 (0.788–3.527)	0.182
Charlson comorbidity index, median (IQR)		4.0 (3.0–6.0)	5.0 (3.0–6.0)	5.0 (3.0–6.0)	1069 (0.951–1.201)	0.262
**APACHE II score, mean (SD)**		**18.4 (7.1)**	**23.8 (8.2)**	**20.6 (8.0)**	**1.099 (1.046–1.154)**	**<0.001**
**Infection type pneumonia (vs. BSI)**		**53 (63)**	**49 (85)**	**102 (72)**	**3.184 (1.378–7.359)**	**0.007**
Pneumonia type (VAP vs. vHAP vs. HAP), n (%)	VAP	41 (77)	40 (82)	81 (79)	Reference	0.861
	vHAP	7 (13)	5 (11)	12 (12)	0.732 (0.215–2.499)	0.619
	HAP	5 (9)	4 (8)	9 (9)	0.820 (0.205–3.276)	0.779
Bacteremic pneumonia, n (%)		28 (53)	28 (57)	56 (55)	1.190 (0.545–2.601)	0.662
Sepsis classification, n (%)	No sepsis	9 (11)	9 (16)	18 (13)	Reference	0.061
	Sepsis	46 (55)	20 (35)	66 (47)	0.435 (0.150–1.258)	0.124
	Septic shock	29 (35)	29 (50)	58 (41)	1.000 (0.347–2.880)	1.000
Vasopressors, n (%)		51 (61)	44 (76)	95 (67)	2.034 (0.966–4.279)	0.061
Invasive mechanical ventilation, n (%)		66 (79)	50 (86)	116 (82)	1.705 (0.686–4.236)	0.251
**CRRT, n (%)**		**6 (7)**	**13 (22)**	**19 (13)**	**3.756 (1.335–10.568)**	**0.012**
Lactate, median (IQR)		1.4 (0.9–1.8)	1.6 (1.0–2.2)	1.4 (0.9–2.0)	1.199 (0.942–1.526)	0.140
**Creatinine, median (IQR)**		**0.8 (0.6–1.3)**	**1.1 (0.7–2.1)**	**0.9 (0.7–1.4)**	**1.946 (1.297–2.918)**	**0.001**
Albumin, mean (SD)		2.9 (0.5)	2.7 (0.5)	2.8 (0.5)	0.513 (0.248–1.059)	0.071
WBC, median (IQR)		13.1 (9.8–16.2)	13.4 (9.9–20.4)	13.2 (9.9–17.6)	1.033 (0.987–1.082)	0.159
**SOFA score, median (IQR)**		**7.5 (4.0–9.0)**	**10.0 (6.0–12.0)**	**8.0 (5.0–10.0)**	**1.124 (1.031–1.224)**	**0.008**
**Empirical treatment receipt, n (%)**		**76 (91)**	**45 (78)**	**121 (85)**	**0.364 (0.140–0.947)**	**0.038**
Active empiric regimen receipt, n (%)		8 (11)	8 (21)	16 (14)	2.065 (0.708–6.017)	0.184
Number of active empiric agents, n (%)		0.0 (0.0–0.0)	0.0 (0.0–0.0)	0.0 (0.0–0.0)	1.813 (0.806–4.077)	0.150
**Sulbactam-containing regimen, n (%)**		**72 (86)**	**41 (71)**	**113 (80)**	**0.402 (0.175–0.924)**	**0.032**
Meropenem-containing regimen, n (%)		51 (61)	27 (47)	78 (55)	0.564 (0.286–1.109)	0.097
Tigecycline-containing regimen, n (%)		18 (21)	18 (31)	36 (25)	1.650 (0.770–3.536)	0.198

**Bold fonts denote statistical significance**. APACHE: Acute Physiology and Chronic Health Evaluation; BSI: Bloodstream infection; CI: Confidence interval; COPD: Chronic obstructive pulmonary disease; COVID-19: Coronavirus disease 2019; CRAB: Carbapenem-resistant *Acinetobacter baumannii*; CRRT: Continuous renal replacement therapy; HAP: Hospital-acquired pneumonia; ICU: Intensive care unit; IQR: Interquartile range; OR: Odds ratio; PDR: Pandrug-resistant; SD: Standard deviation; SOFA: Sequential Organ Failure Assessment; VAP: Ventilator-associated pneumonia; vHAP: Ventilated hospital-acquired pneumonia; WBC: White blood cell count.

**Table 4:** Multivariable logistic regression analysis of clinical failure on day 14 in the primary and propensity score-matched cohorts.

Primary Cohort (N = 142)		
Variable	Odds Ratio (95% CI)	*p*-Value
PDR resistance phenotype	1.147 (0.403–3.437)	0.800
**Admission APACHE II score**	**1.103 (1.038–1.179)**	**0.003**
**Pneumonia (vs. BSI)**	**3.863 (1.372–12.649)**	**0.016**
Serum creatinine	1.497 (0.960–2.545)	0.099
Active empiric regimen	2.535 (0.571–12.254)	0.228
**Sulbactam-containing regimen**	**0.238 (0.065–0.785)**	**0.023**
Propensity score-matched analysis (N = 76)		
PDR resistance phenotype	0.69 (0.26–1.82)	0.451
Admission APACHE II score	1.07 (1.00–1.15)	0.060
Age	1.00 (0.96–1.03)	0.885

**Bold values indicate statistical significance**. APACHE II: Acute Physiology and Chronic Health Evaluation II; BSI: Bloodstream infection; CI: Confidence interval; PDR: Pandrug-resistant.

**Table 5:** Multivariable Cox proportional-hazards analysis of 28-day mortality in the primary and propensity score-matched cohorts.

Primary Cohort (N = 141 ^†^)		
Variable	Hazard Ratio (95% CI)	*p*-Value
Resistance phenotype	2.027 (0.937–4.386)	0.073
**Age**	**1.038 (1.009–1.067)**	**0.010**
**Tigecycline-containing regimen**	**2.094 (1.014–4.323)**	**0.046**
SOFA score	1.248 (1.130–1.379)	<0.001
Lactate	0.519 (0.278–0.974)	0.041
**Lactate** × **time interaction**	**1.321 (1.024–1.705)**	**0.032**
Propensity score-matched analysis (N = 76)		
Resistance phenotype (PDR vs. CRAB)	2.363 (0.969–5.762)	0.059
Admission APACHE II score	1.105 (1.041–1.173)	0.001

^†^ The survival status of 28 days was unknown for one patient. **Bold values indicate statistical significance.** APACHE: Acute Physiology and Chronic Health Evaluation; CI: Confidence interval; CRAB: Carbapenem-resistant *Acinetobacter baumannii*; PDR: Pandrug-resistant; SOFA: Sequential Organ Failure Assessment.

## Data Availability

The datasets generated and/or analysed during the current study are not publicly available because they contain sensitive multicentre clinical information and are subject to institutional and ethical restrictions. De-identified data may be made available by the corresponding author on reasonable request, subject to approval by the participating institutions and compliance with applicable data-protection regulations.

## Supplementary Materials

The following supporting information can be downloaded at: https://www.mdpi.com/article/10.3390/antibiotics15080809/s1, Figure S1: Evolution of SOFA score among survivors in the two resistance phenotypes; Figure S2: Survival curves in the matched cohort (N = 76) according to resistance phenotype; Table S1: Empiric regimens per resistance phenotype; Table S2: Targeted intravenous regimen per resistance phenotype; Table S3: Key antibiotics used as components of targeted intravenous treatment according to resistance phenotype; Table S4: Secondary outcomes; Table S5: Evolution of SOFA score [mean (SD)] among survivors in the two resistance phenotypes; Table S6: Post hoc sensitivity analysis; Table S7: Post hoc sensitivity analysis; Table S8: Univariate Cox regression analysis for the prediction of 28-day mortality; Table S9: Post hoc sensitivity analysis; Table S10: Post hoc sensitivity analysis; Table S11: Logistic regression model used for the prediction of the PDR phenotype; Table S12: Baseline and infection onset characteristics in the matched population; Table S13: Standardized mean differences in the original (SMD-pre) and matched (SMD-post) cohorts; Table S14: Primary and secondary outcomes in the matched cohort; Table S15: Secondary outcomes in the matched cohort.
