# Supplementary material for: Clinical Outcomes of Pandrug-Resistant Versus Carbapenem-Resistant, Colistin-Susceptible Acinetobacter baumannii Infections: A Retrospective Analysis of a Prospective Multicentre Cohort
Source: Antibiotics (Basel). 2026 Aug 19;15(8):809. doi: 10.3390/antibiotics15080809 (PMC13510005; doi:10.3390/antibiotics15080809)

Original cohort

Table S1. Empiric regimens per resistance phenotype.

Regimen	CRAB (N=42)	PDR (N=79)	Total (N=121)
Colistin – Meropenem	5 (12)	13 (17)	18 (15)
Meropenem	2 (5)	14 (18)	16 (13)
Piperacillin/tazobactam	3 (7)	8 (10)	11 (9)
Ceftazidime/avibactam – Colistin	3 (7)	3 (4)	6 (5)
Ampicillin/sulbactam – Colistin	2 (5)	4 (5)	6 (5)
Linezolid – Meropenem	3 (7)	1 (1)	4 (3)
Ceftazidime/avibactam	2 (5)	2 (3)	4 (3)
Ampicillin/sulbactam – Colistin – Meropenem	0 (0)	4 (5)	4 (3)
Vancomycin – Meropenem	1 (2)	2 (3)	3 (3)
Colistin – Tigecycline	1 (2)	2 (3)	3 (3)
Colistin	0 (0)	3 (4)	3 (3)
Moxifloxacin – Piperacillin/tazobactam	2 (5)	0 (0)	2 (2)
Linezolid – Piperacillin/tazobactam	1 (3)	1 (1)	2 (2)
Daptomycin – Meropenem	1 (2)	1 (1)	2 (2)
Daptomycin – Colistin	0 (0)	2 (3)	2 (2)
Colistin – Linezolid – Meropenem	0 (0)	2 (3)	2 (2)
Ampicillin/sulbactam – Colistin – Linezolid – Meropenem	1 (2)	1 (1)	2 (2)
Vancomycin – Piperacillin/tazobactam	1 (2)	0 (0)	1 (1)
Vancomycin – Colistin – Meropenem	0 (0)	1 (1)	1 (1)
Vancomycin	0 (0)	1 (1)	1 (1)
Tigecycline	1 (2)	0 (0)	1 (1)
Other	1 (2)	0 (0)	1 (1)
Meropenem – Piperacillin/tazobactam	0 (0)	1 (1)	1 (1)
Meropenem – Other	1 (2)	0 (0)	1 (1)
Linezolid – Meropenem/vaborbactam	0 (0)	1 (1)	1 (1)
Linezolid	1 (2)	0 (0)	1 (1)
Gentamicin – Meropenem	1 (2)	0 (0)	1 (1)
Doxycycline	0 (0)	1 (1)	1 (1)
Daptomycin – Tigecycline	0 (0)	1 (1)	1 (1)
Daptomycin – Meropenem/vaborbactam	1 (2)	0 (0)	1 (1)
Colistin – Rifampin	0 (0)	1 (1)	1 (1)
Colistin – Other	0 (0)	1 (1)	1 (1)
Colistin – Meropenem – Minocycline	0 (0)	1 (1)	1 (1)
Colistin – Linezolid – Meropenem – Metronidazole	0 (0)	1 (1)	1 (1)
Ceftriaxone – Clindamycin	0 (0)	1 (1)	1 (1)
Ceftriaxone	1 (2)	0 (0)	1 (1)
Ceftazidime/avibactam – Piperacillin/tazobactam	1 (2)	0 (0)	1 (1)

**Regimen**	**CRAB (N=42)**	**PDR (N=79)**	**Total (N=121)**
Ceftazidime/avibactam – Colistin – Tigecycline	0 (0)	1 (1)	1 (1)
Cefepime – Linezolid	1 (2)	0 (0)	1 (1)
Ampicillin/sulbactam – Vancomycin – Colistin – Meropenem	0 (0)	1 (1)	1 (1)
Ampicillin/sulbactam – Gentamicin – Colistin iv – Colistin neb – Fosfomycin	1 (2)	0 (0)	1 (1)
Ampicillin/sulbactam – Colistin – Tigecycline	0 (0)	1 (1)	1 (1)
Ampicillin/sulbactam – Colistin -Moxifloxacin	1 (2)	0 (0)	1 (1)
Ampicillin/sulbactam – Colistin iv – Colistin neb – Other	1 (2)	0 (0)	1 (1)
Ampicillin/sulbactam – Colistin iv – Colistin neb – Meropenem – Piperacillin/tazobactam – Other	1 (2)	0 (0)	1 (1)
Ampicillin/sulbactam	1 (2)	0 (0)	1 (1)
Amikacin – Vancomycin – Meropenem	0 (0)	1 (1)	1 (1)
Amikacin – Piperacillin/tazobactam	0 (0)	1 (1)	1 (1)

CRAB: Carbapenem-resistant *Acinetobacter baumannii;* PDR: Pandrug-resistant.

Table S2. Targeted intravenous regimen per resistance phenotype.

Regimen	Total	PDR (n=91)	CRAB (n=51)
Colistin-Ampicillin/sulbactam-Meropenem	65 (46)	61 (67)	4 (8)
Colistin-Ampicillin/sulbactam-Tigecycline	29 (20)	25 (27)	4 (8)
Colistin-Ampicillin/sulbactam	19 (13)	0 (0)	19 (37)
Colistin-Meropenem	8 (6)	0 (0)	8 (16)
Colistin	6 (4)	0 (0)	6 (12)
Colistin-Tigecycline	4 (3)	1 (1)	3 (6)
Colistin-Meropenem-Tigecycline	2 (1)	1 (1)	1 (2)
Colistin-Other	2 (1)	0 (0)	2 (4)
Colistin-Rifampin	2 (1)	1 (1)	1 (2)
Colistin-Vancomycin	2 (1)	0 (0)	2 (4)
Colistin-Meropenem-Daptomycin-Gentamicin	1 (1)	1 (1)	0 (0)
Colistin-Meropenem-Gentamicin	1 (1)	0 (0)	1 (2)
Meropenem-Tigecycline	1 (1)	1 (1)	0 (0)

CRAB: Carbapenem-resistant *Acinetobacter baumannii*; PDR: Pandrug-resistant.

Table S3. Key antibiotics used as components of targeted intravenous treatment according to resistance phenotype.

Antibiotic	Total	CRAB	PDR	p-value
Colistin-containing regimen	141 (99)	51 (100)	90 (99)	0.452
Tigecycline-containing regimen	36 (25)	8 (16)	28 (30)	0.047
Meropenem-containing regimen	78 (55)	14 (28)	64 (70)	<0.001
Sulbactam-containing regimen	113 (80)	27 (53)	86 (95)	<0.001

CRAB: Carbapenem-resistant *Acinetobacter baumannii*; PDR: Pandrug-resistant.

Table S4. Secondary outcomes

Outcome	Total	CRAB	PDR	p-value
Microbiological eradication in the microbiologically evaluable population (N=127)	104 (82)	40 (89)	64 (78)	0.129
BSI (N=38)	37 (97)	17 (100)	20 (95)	0.362
VAP/HAP/vHAP (N=89)	67 (75)	23 (82)	44 (72)	0.309
Recurrent infection, n (%)	9 (6)	3 (6)	6 (7)	0.867
Superinfection, n (%)	17 (12)	6 (12)	11 (12)	0.955
Day-28 mortality† (N=141), n (%)	41 (29)	11 (22)	30 (33)	0.139
**Intervention-free days in the ICU population (N=128, CRAB N=51, PDR N=77)**				
ICU-free days	0.0 (-1.0-9.0)	0.0 (0.0-6.0)	0.0 (-1.0-9.0)	0.498
Ventilator-free days	1.0 (-1.0-19.0)	1.0 (0.0-19.0)	5.0 (-1.0-22.0)	0.812
Vasopressor-free days	12.0 (0.0-23.0)	12.0 (-1.0-24.0)	12.0 (-1.0-23.5)	0.994
CRRT-free days	28.0 (-1.0-28.0)	28.0 (8.0-28.0)	28.0 (-1.0-28.0)	0.430
Organ support-free days	0.0 (-1.0-17.5)	0.0 (0.0-15.0)	1.0 (-1.0-18.0)	0.903

† The survival status at day 28 was unknown for one patient. BSI: Bloodstream infection; CRAB: Carbapenem-resistant *Acinetobacter baumannii*; CRRT: Continuous renal replacement therapy; HAP: Hospital-acquired pneumonia; ICU: Intensive care unit; PDR: Pandrug-resistant; VAP: Ventilator-associated pneumonia; vHAP: Ventilated hospital-acquired pneumonia.

Table S5. Evolution of SOFA score [mean (SD)] among survivors in the two resistance phenotypes.

	Total	CRAB	PDR
Infection Onset	8.13 (4.19)	8.57 (3.47)	7.89 (4.55)
Day 7	6.83 (4.60)	7.22 (4.24)	6.61 (4.80)
Day 14	5.52 (4.24)	6.00 (3.86)	5.23 (4.46)

Table S6. Post hoc sensitivity analysis. The logistic regression analysis on day 14 clinical failure was performed in a population of 116 subjects, whose isolates were tested for colistin susceptibility via broth microdilution.

Variable	Odds ratio (95% CI)	p-value
PDR resistance phenotype	0.679 (0.180-2.646)	0.567
**Admission APACHE II score**	**1.115 (1.039-1.206)**	**0.004**
**Pneumonia (vs BSI)**	**4.699 (1.360-21.052)**	**0.024**
Serum creatinine	1.466 (0.915-2.586)	0.140
Active empiric regimen	1.728 (0.255-13.028)	0.579
Sulbactam-containing regimen	0.222 (0.048-1.003)	0.055

APACHE, Acute Physiology and Chronic Health Evaluation; BSI, Bloodstream infections; PDR, Pandrug-resistant

Table S7. *Post hoc sensitivity analysis. The logistic regression analysis on day 14 clinical failure was performed in a population of 113 subjects who did not concurrently receive ampicillin-sulbactam and tigecycline as targeted treatment.*

Variable	Odds ratio (95% CI)	p-value
PDR resistance phenotype	0.618 (0.187-2.049)	0.432
**Admission APACHE II score**	**1.123 (1.043-1.209)**	**0.002**
**Pneumonia (vs BSI)**	**3.873 (0.984-15.240)**	**0.053**
**Serum creatinine**	**1.774 (1.020-3.084)**	**0.042**
Active empiric regimen	2.735 (0.508-14.717)	0.241
**Sulbactam-containing regimen**	**0.235 (0.063-0.882)**	**0.032**

APACHE, Acute Physiology and Chronic Health Evaluation; BSI, Bloodstream infections; PDR, Pandrug-resistant

Table S8. Univariate Cox regression analysis for the prediction of 28-day mortality.

Variable		Died (n=41)	Survived (n=100)	Total (n=141†)	HR (95% CI)	p-value
CRAB phenotype, n (%)		11 (27)	40 (40)	51 (36)	0.604 (0.303-1.206)	0.153
PDR phenotype, n (%)		30 (73)	60 (60)	90 (64)		
**Age**, median (IQR)		**75 (67-84)**	**67 (58-73)**	**69 (60-77)**	**1.033 (1.007-1.060)**	**0.014**
Male gender, n (%)		23 (56)	60 (60)	83 (59)	1.097 (0.592-2.033)	0.769
ICU stay (vs ward), n (%)		37 (90)	91 (91)	128 (91)	1.077 (0.384-3.022)	0.888
COVID-19, n (%)		6 (15)	12 (12)	18 (13)	1.220 (0.513-2.901)	0.653
Smoking, n (%)		14 (41)	46 (55)	60 (51)	0.623 (0.314-1.233)	0.174
Immunosuppression, n (%)		5 (12)	13 (13)	18 (13)	0.904 (0.355-2.304)	0.833
	**Non-fatal disease**	**21 (51)**	**77 (78)**	**98 (70)**	**Reference**	**0.013**

Variable		Died (n=41)	Survived (n=100)	Total (n=141†)	HR (95% CI)	p-value
**McCabe-Jackson**, n (%)	**Ultimately fatal disease**	**17 (42)**	**16 (16)**	**33 (24)**	**2.622 (1.382-4.974)**	**0.003**
	Rapidly fatal disease	3 (7)	6 (6)	9 (6)	1.638 (0.488-5.491)	0.424
Known XDR/PDR Acinetobacter colonization, n (%)		2 (5)	13 (13)	15 (11)	0.386 (0.093-1.600)	0.190
Diabetes, n (%)	None	30 (73)	72 (72)	102 (72)	Reference	0.801
	Uncomplicated	4 (10)	16 (16)	20 (14)	0.633 (0.223-1.798)	0.391
	End-organ damage	7 (17)	12 (12)	19 (14)	1.354 (0.594-3.083)	0.471
Malignancy, n (%)	None	34 (83)	78 (79)	113 (80)	Reference	0.801
	Any leukemia/lymphoma or localized solid tumor	5 (12)	15 (15)	20 (14)	0.754 (0.295-1.929)	
	Metastatic solid	2 (5)	6 (6)	8 (6)	0.794 (0.191-3.305)	
Congestive heart failure, n (%)		9 (22)	14 (14)	23 (16)	1.550 (0.739-3.247)	0.246
COPD, n (%)		11 (27)	27 (27)	38 (27)	0.946 (0.474-1.888)	0.876
**Charlson comorbidity index**, median (IQR)		**6.0 (4.0-7.0)**	**4.0 (3.0-6.0)**	**5.0 (3.0-6.0)**	**1.127 (1.019-1.247)**	**0.021**
**APACHE II score**, mean (SD)		**25.0 (8.1)**	**18.8 (7.3)**	**20.6 (8.0)**	**1.093 (1.050-1.138)**	**<0.001**
BSI (vs pneumonia), n (%)		13 (32)	27 (27)	40 (28)	0.845 (0.438-1.631)	0.616
BSI type, n (%)	Primary	10 (77)	17 (63)	27 (68)	Reference	0.146
	CRBSI	1 (8)	9 (33)	10 (25)	0.220 (0.028-1.717)	0.149
	Secondary	2 (15)	1 (4)	3 (8)	2.452 (0.529-11.357)	0.252
Pneumonia type, n (%)	VAP	20 (71)	60 (82)	80 (79)	Reference	0.417
	vHAP	5 (18)	7 (10)	12 (12)	1.844 (0.692-4.915)	0.221
	HAP	3 (11)	6 (8)	9 (9)	1.535 (0.456-5.169)	0.489
Bacteremic pneumonia, n (%)		16 (57)	40 (55)	56 (55)	1.130 (0.535-2.389)	0.749
Time to AST reporting, median (IQR)		2.0 (2.0-3.0)	2.0 (2.0-3.0)	2.0 (2.0-3.0)	0.997 (0.787-1.264)	0.983
**Sepsis level**, n (%)	**No sepsis**	**3 (7)**	**14 (14)**	**17 (12)**	**Reference**	**<0.001**
	Sepsis	10 (24)	56 (56)	66 (47)	0.860 (0.237-3.126)	0.819
	**Septic shock**	**28 (68)**	**30 (30)**	**58 (41)**	**3.433 (1.043-11.299)**	**0.042**
Vasopressors, n (%)		32 (78)	62 (62)	94 (67)	1.905 (0.909-3.992)	0.088
Respiratory rate, median (IQR)		24 (22-28)	24 (18-28)	24 (19-28)	1.022 (0.977-1.069)	0.337
Highest temperature, mean (SD)		37.6 (1.2)	37.9 (0.8)	37.8 (0.9)	0.741 (0.534-1.028)	0.073

Variable	Died (n=41)	Survived (n=100)	Total (n=141†)	HR (95% CI)	p-value
Lowest SAP, median (IQR)	102 (90-109)	104 (94-110)	103 (93-110)	0.994 (0.980-1.008)	0.414
Highest heart rate, mean (SD)	105 (22)	100 (16)	101 (18)	1.015 (0.998-1.033)	0.082
Invasive mechanical ventilation, n (%)	35 (85)	80 (80)	115 (82)	1.391 (0.585-3.308)	0.455
**CRRT**, n (%)	**12 (29)**	**7 (7)**	**19 (14)**	**3.664 (1.861-7.211)**	**<0.001**
PaO2, median (IQR)	83 (73-96)	80 (73-98)	81 (73-98)	1.004 (0.991-1.017)	0.591
PaCO2, median (IQR)	42 (36-49)	41 (36-48)	41 (36-48)	1.007 (0.978-1.037)	0.640
**pH**, median (IQR)	**7.40 (7.29-7.45)**	**7.43 (7.38-7.47)**	**7.42 (7.37-7.47)**	**0.003 (0.000-0.090)**	**0.001**
**Lactate**, median (IQR)	**2.0 (1.4-2.9)**	**1.1 (0.9-1.8)**	**1.4 (0.9-2.0)**	**1.346 (1.194-1.518)**	**<0.001**
**Urea**, median (IQR)	**80 (65-128)**	**49 (33-96)**	**66 (38-104)**	**1.008 (1.003-1.013)**	**0.001**
**Creatinine**, median (IQR)	**1.4 (0.8-1.9)**	**0.8 (0.6-1.2)**	**0.9 (0.7-1.4)**	**1.402 (1.133-1.735)**	**0.002**
**Bilirubin**, median (IQR)	**0.8 (0.6-1.8)**	**0.6 (0.4-0.8)**	**0.6 (0.4-1.0)**	**1.077 (1.034-1.123)**	**<0.001**
**SGOT**, median (IQR)	**45 (25-78)**	**33 (22-53)**	**34 (23-58)**	**1.001 (1.000-1.002)**	**0.001**
**SGPT**, median (IQR)	**42 (24-75)**	**33 (18-51)**	**33 (19-57)**	**1.001 (1.000-1.002)**	**0.023**
Albumin, mean (SD)	2.7 (0.5)	2.9 (0.5)	2.8 (0.5)	0.554 (0.285-1.076)	0.081
**Prothrombin time**, median (IQR)	**14.0 (12.8-15.6)**	**13.1 (11.6-14.8)**	**13.5 (11.7-15.3)**	**1.122 (1.000-1.258)**	**0.050**
**aPTT**, median (IQR)	**39 (36-48)**	**33 (31-39)**	**35 (31-42)**	**1.038 (1.016-1.061)**	**0.001**
HgB, median (IQR)	9.4 (8.4-10.4)	9.2 (8.5-10.6)	9.2 (8.4-10.5)	1.035 (0.911-1.177)	0.595
**PLT**, median (IQR)	**204 (86-284)**	**259 (171-362)**	**244 (150-332)**	**0.996 (0.993-0.999)**	**0.003**
WBC, median (IQR)	13.2 (10.0-20.2)	13.5 (9.5-16.7)	13.3 (9.9-17.6)	1.007 (0.971-1.044)	0.702
**Neutrophils**, median (IQR)	**87.6 (81.9-93.4)**	**84.0 (76.8-89.0)**	**85.0 (77.0-91.0)**	**1.053 (1.009-1.099)**	**0.017**
Respiratory SOFA, median (IQR)	2.6 (1.0)	2.3 (0.9)	2.4 (0.9)	1.329 (0.925-1.909)	0.123
**Hemodynamic SOFA**, median (IQR)	**2.8 (1.6)**	**2.1 (1.7)**	**2.3 (1.7)**	**1.272 (1.026-1.577)**	**0.028**
**Liver SOFA**, median (IQR)	**0.9 (1.4)**	**0.2 (0.5)**	**0.4 (0.9)**	**1.674 (1.331-2.106)**	**<0.001**
**Coagulation SOFA**, median (IQR)	**0.8 (1.3)**	**0.3 (0.6)**	**0.4 (0.9)**	**1.594 (1.230-2.064)**	**<0.001**
**Brain SOFA**, median (IQR)	**2.4 (1.6)**	**1.7 (1.4)**	**1.9 (1.5)**	**1.355 (1.095-1.676)**	**0.005**
**Renal SOFA**, median (IQR)	**1.2 (1.4)**	**0.5 (1.0)**	**0.7 (1.1)**	**1.432 (1.158-1.770)**	**0.001**

Variable	Died (n=41)	Survived (n=100)	Total (n=141†)	HR (95% CI)	p-value
**SOFA score**, median (IQR)	**10.0 (8.0-14.0)**	**7.0 (4.0-9.0)**	**8.0 (5.0-10.0)**	**1.178 (1.105-1.256)**	**<0.001**
Empiric treatment receipt, n (%)	36 (88)	84 (84)	120 (85)	1.292 (0.507-3.292)	0.592
Active empiric regimen, n (%)	3 (10)	13 (17)	16 (15)	0.579 (0.176-1.906)	0.369
Number of active empiric antibiotics	0 (0-0)	0 (0-0)	0 (0-0)	0.519 (0.184-1.462)	0.214
Sulbactam containing regimen (N=141) , n (%)	33 (81)	79 (79)	112 (79.4)	1.043 (0.482-2.259)	0.914
Meropenem containing regimen (N=141) , n (%)	20 (49)	58 (58)	78 (55)	0.755 (0.409-1.392)	0.368
**Tigecycline containing regimen (N=141)** , n (%)	**17 (42)**	**18 (18)**	**35 (25)**	**2.529 (1.357-4.713)**	**0.003**

† Survival status at day 28 was unknown for one patient. Bold values indicate statistical significance. APACHE: Acute Physiology and Chronic Health Evaluation; BSI: Bloodstream infection; CI: Confidence interval; COPD: Chronic obstructive pulmonary disease; COVID-19: Coronavirus disease 2019; CRAB: Carbapenem-resistant *Acinetobacter baumannii*; CRRT: Continuous renal replacement therapy; HAP: Hospital-acquired pneumonia; HR: Hazard ratio; ICU: Intensive care unit; IQR: Interquartile range; PDR: Pandrug-resistant; PLT: Platelets; SD: Standard deviation; SOFA: Sequential Organ Failure Assessment; VAP: Ventilator-associated pneumonia; vHAP: Ventilated hospital-acquired pneumonia; WBC: White blood cell count; XDR: Extensively drug-resistant.

Table S9. Post hoc sensitivity analysis. The Cox regression analysis on 28-day all-cause mortality was performed in a population of 116 subjects, whose isolates were tested for colistin susceptibility via broth microdilution.

**Variable**	**Hazard Ratio (95% CI)**	**p-value**
PDR Resistance Phenotype	1.545 (0.677-3.527)	0.302
**Age**	**1.040 (1.009-1.072)**	**0.012**
Tigecycline containing regimen	2.044 (0.943-4.426)	0.070
**SOFA score**	**1.243 (1.123-1.378)**	**<0.001**
**Lactate**	**0.441 (0.199-0.977)**	**0.044**
**Lactate*time interaction**	**1.374 (1.009-1.871)**	**0.044**

PDR: Pandrug-resistant; SOFA: Sequential Organ Failure Assessment.

Table S10. Post hoc sensitivity analysis. The Cox regression analysis on day 28 all-cause mortality was performed in a population of 113 subjects who did not concurrently receive ampicillin-sulbactam and tigecycline as targeted treatment.

**Variable**	**Hazard ratio (95% CI)**	**p-value**
PDR resistance phenotype	1.432 (0.599-3.424)	0.419
**Age**	**1.054 (1.017-1.092)**	**0.004**
Tigecycline-containing regimen	2.897 (0.925-9.071)	0.068
**SOFA score**	**1.225 (1.100-1.364)**	**<0.001**
Lactate	1.015 (0.611-1.687)	0.954
Lactate*time interaction	1.03 (0.985-1.068)	0.220

PDR: Pandrug-resistant; SOFA: Sequential Organ Failure Assessment.

Figure S1. Evolution of SOFA score among survivors in the two resistance phenotypes. CRAB phenotype was associated with a non-significant decrease in mean SOFA score between infection onset and day 7 (1.344 [−0.751–3.439], p = 0.371) and a significant decrease between infection onset and day 14 (2.569 [0.372–4.765], p = 0.016). PDR phenotype was associated with a non-significant decrease in mean SOFA score between infection onset and day 7 (1.283 [−0.302–2.868], p = 0.157) and a significant decrease between infection onset and day 14 (2.658 [0.987–4.330], p < 0.001). Mean SOFA score did not differ significantly between resistance phenotypes at any time point. In the linear mixed model, time point, but not resistance phenotype or the resistance phenotype-by-time interaction, was a significant determinant of SOFA score.

CRAB: Carbapenem-resistant *Acinetobacter baumannii*; PDR: Pandrug-resistant; SOFA: Sequential Organ Failure Assessment.

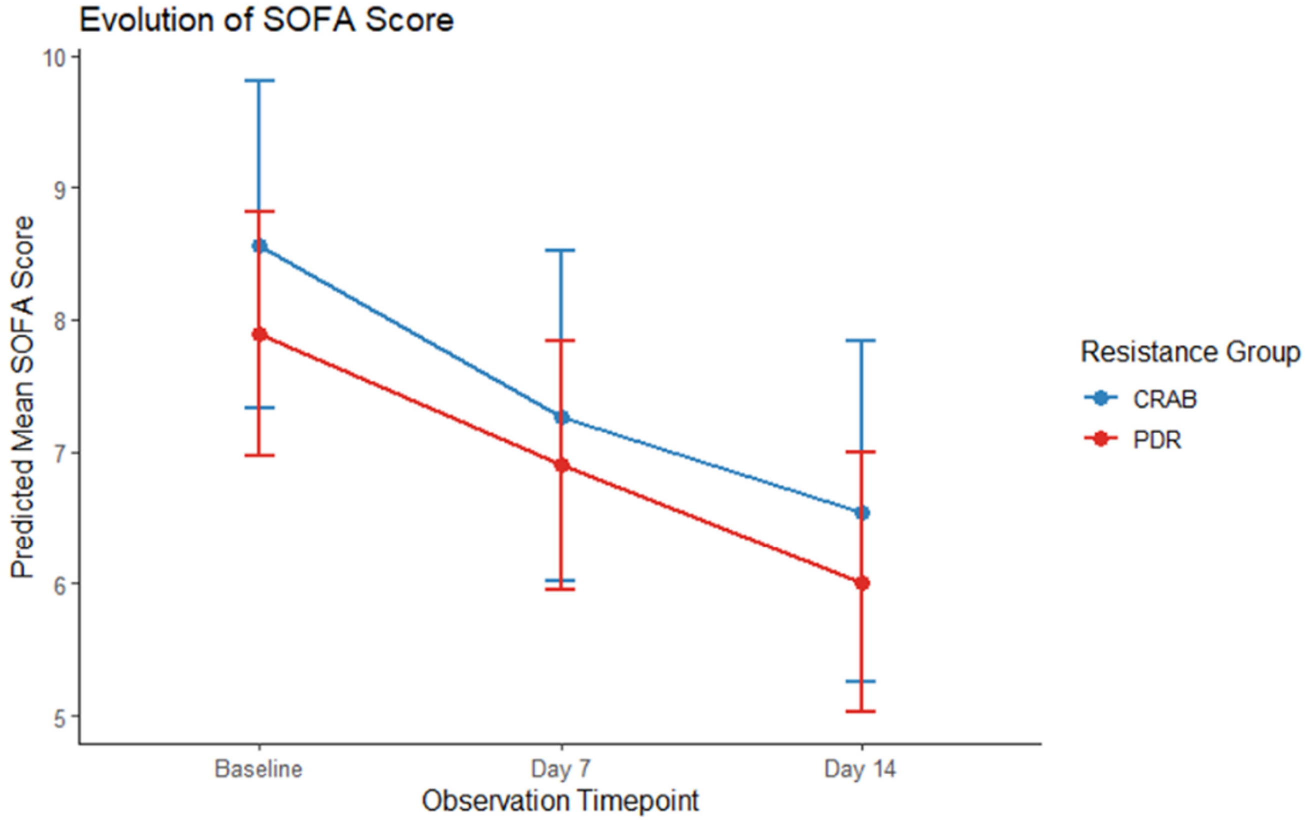

Matching procedure and matched cohorts

Table S11. Logistic regression model used for the prediction of the PDR phenotype. The probability of belonging to the PDR phenotype, derived from this model, was used for the nearest-neighbour propensity score matching.

**Variable**		**OR (95%) CI**	**p-value**
Patient disposition (ICU vs ward)		9.59 10e8 (0.0- ∞)	0.998
Sepsis level	No sepsis	Reference	0.102
	Sepsis	0.112 (0.013-0.945)	0.044
	Septic shock	0.179 (0.020-1.598)	0.124
Infection onset SOFA score		1.001 (0.880-1.138)	0.993
Infection type	BSI	Reference	0.353
	VAP	1.713 (0.716-4.102)	0.227
	vHAP	1.522 (0.359-6.464)	0.569
	HAP	4.392 (0.756-25.501)	0.099
McCabe-Jackson classification	No fatal disease	Reference	0.2368
	Ultimately fatal disease	0.449 (0.178-1.134)	0.090
	Rapidly fatal disease	0.883 (0.168-4.637)	0.883
Admission APACHE II score		1.023 (0.966-1.084)	0.435

APACHE: Acute Physiology and Chronic Health Evaluation; BSI: Bloodstream Infection; HAP: Hospital-Acquired Pneumonia; ICU: Intensive Care Unit; PDR: Pandrug-resistant; SOFA: Sepsis-related Organ Function Assessment; VAP: Ventilator-Associated Pneumonia; vHAP: ventilated Hospital-Acquired Pneumonia

Table S12. Baseline and infection onset characteristics in the matched population.

Variable		Total (N=76)	CRAB (n=38)	PDR (n=38)	p-value	SMD
Age, median (IQR)		67 (58-75)	68 (60-78)	67 (56-73)	0.540	0.202
Male gender, n (%)		45 (59)	21 (55)	24 (63)	0.484	0.161
In ICU at enrollment, n (%)		76 (100)	38 (100)	38 (100)	N/A	0.000
COVID-19 admission, n (%)		9 (12)	4 (11)	5 (13)	0.723	0.082
Immunosuppression, n (%)		10 (13)	5 (13)	5 (13)	1.000	0.000
Diabetes mellitus, n (%)		17 (22)	8 (21)	9 (24)	0.730	0.183
Malignancy, n (%)		13 (17)	8 (21)	5 (13)	0.327	0.348
Moderate-severe CKD, n (%)		8 (11)	3 (8)	5 (13)	0.455	0.172
Congestive heart failure, n (%)		12 (16)	6 (16)	6 (16)	1.000	0.000
Coronary heart disease, n (%)		15 (20)	6 (16)	9 (24)	0.387	0.199
COPD, n (%)		22 (29)	10 (26)	12 (32)	0.613	0.116
McCabe-Jackson classification, n (%)	Non-fatal disease	54 (71)	26 (68)	28 (74)	0.584	0.239
	Ultimately fatal disease	18 (24)	9 (24)	9 (24)		
	Rapidly fatal disease	4 (5)	3 (8)	1 (3)		
Charlson comorbidity index, median (IQR)		4 (3-6)	4 (3-6)	4 (2-6)	0.896	0.068
Admission APACHE II score, mean (SD)		20.9 (7.5)	21.7 (7.8)	20.1 (7.3)	0.332	0.224
Infection type, n (%)	BSI	27 (36)	15 (39)	12 (32)	0.472	0.167
	HAP/VAP	49 (64)	23 (61)	26 (69)		
Time from ICU admission (days, N=75), median (IQR)		9 (4-14)	9 (4-13)	8 (6-20)	0.517	0.355
Pneumonia type, n (%) (N=49)	VAP	42 (86)	19 (83)	23 (89)	0.819	0.180
	vHAP	5 (10)	3 (13)	2 (8)		
	HAP	2 (4)	1 (4)	1 (4)		
Bacteremic pneumonia, n (%) (N=49)		29 (59)	13 (57)	16 (62)	0.721	0.102
Sepsis, n (%)		38 (50)	19 (50)	19 (50)	1.000	0.000
Septic shock, n (%)		36 (47)	18 (47)	18 (47)		
Vasopressor use, n (%)		61 (80)	33 (87)	28 (74)	0.150	0.335
Invasive MV, n (%)		73 (96)	37 (97)	36 (95)	0.556	0.136
CRRT, n (%)		10 (13)	4 (11)	6 (16)	0.497	0.156
SOFA score, median (IQR)		8.0 (6.0-10.8)	8.5 (7.0-11.0)	8.0 (6.0-10.0)	0.439	0.095

Variable		Total (N=76)	CRAB (n=38)	PDR (n=38)	p-value	SMD
Lactate (mEq/L), median (IQR) (N=75)		1.4 (1.0-2.0)	1.6 (0.9-2.2)	1.4 (1.0-1.7)	0.420	0.071
Creatinine (mg/dL), median (IQR)		0.9 (0.6-1.4)	0.9 (0.6-1.4)	1.0 (0.6-1.4)	0.971	0.010
Albumin (g/dL), mean (SD) (N=68)		2.8 (0.5)	2.7 (0.5)	2.9 (0.5)	0.163	0.342
WBC (*103/μL), median (IQR)		13.7 (9.9-20.2)	13.8 (9.9-20.0)	13.4 (9.9-20.2)	0.884	0.161
Empirical treatment receipt, n (%)		68 (90)	32 (84)	36 (95)	0.135	0.348
Number of empirical antimicrobials per patient (median, IQR)		2 (1-2)	2 (1-2)	2 (1-2)	0.251	0.118
Duration of empiric treatment per antibiotic (days), median (IQR)		3.0 (2.0-3.5)	3.0 (2.0-4.0)	3.0 (2.0-3.0)	0.700	0.065
Number of active empirical antimicrobials per patient (N=68), n (%)	0	55 (81)	19 (59)	36 (100)	<0.001	1.170
	1	10 (15)	10 (31)	0 (0)		
	2	3 (4)	3 (9)	0 (0)		

McCabe–Jackson classification follows European Centre for Disease Prevention and Control guidance: non-fatal, ultimately fatal, and rapidly fatal disease correspond to expected survival of ≥5 years, 1–5 years, and <1 year, respectively. APACHE II, Acute Physiology and Chronic Health Evaluation II; BSI, bloodstream infection; CKD, chronic kidney disease; COPD, chronic obstructive pulmonary disease; COVID-19, coronavirus disease 2019; CRAB, Carbapenem-Resistant *Acinetobacter baumannii*; CRRT, continuous renal replacement therapy; HAP, hospital-acquired pneumonia; ICU, intensive care unit; IQR, interquartile range; MV, mechanical ventilation; PDR, Pandrug-resistant; SD, standard deviation; SMD, standardized mean difference; SOFA, Sequential Organ Failure Assessment; VAP, ventilator-associated pneumonia; vHAP, ventilated hospital-acquired pneumonia; WBC, white blood cell count.

Table S13. Standardized mean differences in the original (SMD-pre) and matched (SMD-post) cohorts.

Variable		SMD-pre	SMD-post
Age, median (IQR)		0.030	0.202
Male gender, n (%)		0.050	0.161
In ICU at enrollment, n (%)		0.577	0.000
COVID-19 admission, n (%)		0.237	0.082
Immunosuppression, n (%)		0.139	0.000
Diabetes mellitus, n (%)		0.174	0.183
Malignancy, n (%)		0.075	0.348
Moderate-severe CKD, n (%)		0.073	0.172
Congestive heart failure, n (%)		0.061	0.000
Coronary heart disease, n (%)		0.064	0.199
COPD, n (%)		0.024	0.116
McCabe-Jackson classification, n (%)	Non-fatal disease	0.238	0.239
	Ultimately fatal disease		
	Rapidly fatal disease		
Charlson comorbidity index, median (IQR)		0.069	0.068
Admission APACHE II score, mean (SD)		0.088	0.224
Infection type, n (%)	BSI	0.178	0.167
	HAP/VAP		
Time from ICU admission (days, N=75), median (IQR)		0.236	0.355
Pneumonia type, n (%) (N=49)	VAP	0.345	0.180
	vHAP		
	HAP		
Bacteremic pneumonia, n (%) (N=49)		0.237	0.102
Sepsis, n (%)		0.579	0.000
Septic shock, n (%)			
Vasopressor use, n (%)		0.471	0.335
Invasive MV, n (%)		0.659	0.136
CRRT, n (%)		0.016	0.156
SOFA score, median (IQR)		0.168	0.095
Lactate (mEq/L), median (IQR) (N=75)		0.044	0.071
Creatinine (mg/dL), median (IQR)		0.096	0.010

<table>
<tr><th colspan="2">Variable</th><th>SMD-pre</th><th>SMD-post</th></tr>
<tr><td colspan="2">Albumin (g/dL), mean (SD) (N=68)</td><td>0.139</td><td>0.342</td></tr>
<tr><td colspan="2">WBC (*103/μL), median (IQR)</td><td>0.162</td><td>0.161</td></tr>
<tr><td colspan="2">Empirical treatment receipt, n (%)</td><td>0.124</td><td>0.348</td></tr>
<tr><td colspan="2">Number of empirical antimicrobials per patient (median, IQR)</td><td>0.018</td><td>0.118</td></tr>
<tr><td colspan="2">Duration of empiric treatment per antibiotic (days), median (IQR)</td><td>0.073</td><td>0.065</td></tr>
<tr><td rowspan="3">Number of active empirical antimicrobials per patient (N=68), n (%)</td><td>0</td><td rowspan="3">1.109</td><td rowspan="3">1.170</td></tr>
<tr><td>1</td></tr>
<tr><td>2</td></tr>
</table>

McCabe–Jackson classification follows European Centre for Disease Prevention and Control guidance: non-fatal, ultimately fatal, and rapidly fatal disease correspond to expected survival of ≥5 years, 1–5 years, and <1 year, respectively. APACHE II, Acute Physiology and Chronic Health Evaluation II; BSI, bloodstream infection; CKD, chronic kidney disease; COPD, chronic obstructive pulmonary disease; COVID-19, coronavirus disease 2019; CRRT, continuous renal replacement therapy; HAP, hospital-acquired pneumonia; ICU, intensive care unit; IQR, interquartile range; MV, mechanical ventilation; SD, standard deviation; SMD, standardized mean difference; SOFA, Sequential Organ Failure Assessment; VAP, ventilator-associated pneumonia; vHAP, ventilated hospital-acquired pneumonia; WBC, white blood cell count.

Table S14. Primary and secondary outcomes in the matched cohort.

Variable		Total (N=76)	CRAB (N=38)	PDR (N=38)	p-value
**Microbiological outcome**					
Positive follow-up blood cultures (days 6-14) (N=26)		0 (0)	0 (0)	0 (0)	N/A
Follow-up respiratory cultures in pneumonia patients (N=49), n (%)	Taken	36 (74)	17 (74)	19 (73)	0.330
	No, presumed eradication	6 (12)	2 (9)	4 (15)	
	Not taken due to moribund condition before day 6	4 (8)	1 (4)	3 (12)	
	Not taken in error	1 (2)	1 (4)	0 (0)	
	Unknown	2 (4)	2 (9)	0 (0)	
Positive follow-up respiratory cultures (days 6-14), (N=36), n (%, phenotype)		8 (22),<br>4 (50, PDR)<br>4 (50, CRAB)	2 (12),<br>0 (0, PDR)<br>2 (100, CRAB)	6 (31),<br>4 (67, PDR)<br>2 (33, CRAB)	0.153
**Clinical outcome**					
Clinical failure on day 14, n (%)		28 (37)	16 (42)	12 (32)	0.342
**Elements of clinical failure on day 14**					
Death up to day 14, n (%)		10 (13)	3 (8)	7 (18)	0.309
**Antimicrobial treatment modification due to clinical deterioration, n (%)**		**9 (12)**	**9 (24)**	**0 (0)**	**0.001**
Antimicrobial withdrawal due to toxicity, n (%)		5 (7)	3 (8)	2 (5)	0.365
Persistent BSI, (N=26), n (%)		0 (0)	0 (0)	0 (0)	N/A
Intubation of initially spontaneously breathing patients (N=2), n (%)		1 (50)	0 (0)	1 (100)	1.000
No improvement in oxygenation (N=37), n (%)		9 (23)	6 (29)	3 (17)	0.464
**Secondary evaluation of clinical outcome**					
Indeterminate outcome, n (%)		11 (15)	6 (16)	5 (13)	0.744
Recurrent infection, n (%)		5 (7)	3 (8)	2 (5)	0.644
Superinfection, n (%)		11 (15)	4 (11)	7 (18)	0.328
**Mortality**					
Day 28 mortality, n (%)		22 (29)	8 (21)	14 (37)	0.129

Bold values indicate statistically significant differences. CRAB: Carbapenem-Resistant *Acinetobacter baumannii*, PDR: Pandrug-resistant; BSI: Bloodstream Infection; N/A: Not Applicable.

Table S15. Secondary outcomes in the matched cohort.

	**Total (N=76)**	**CRAB (N=38)**	**PDR (N=38)**	**p-value**
Microbiological eradication in the microbiologically evaluable population (N=68)	60 (88)	32 (94)	28 (82)	0.132
BSI (N=26)	26 (100)	15 (100)	11 (100)	N/A
VAP/HAP/vHAP (N=42)	34 (81)	17 (90)	17 (74)	0.201
Recurrent infection, n (%)	5 (7)	3 (8)	2 (5)	0.644
Superinfection, n (%)	11 (15)	4 (11)	7 (18)	0.328
Day 28 mortality, n (%)	22 (29)	8 (21)	14 (37)	0.129
**Intervention-free days in the ICU population**				
ICU-free days	0.0 (-1.0-6.5)	0.0 (0.0-6.0)	0.0 (-1.0-7.0)	0.284
Ventilator-free days	0.5 (-1.0-18.5)	1.0 (0.0-19.0)	0.0 (-1.0-18.0)	0.649
Vasopressor-free days	11.0 (-1.0-23.0)	11.0 (0.0-22.0)	9.0 (-1.0-23.0)	0.666
CRRT-free days	28.0 (-1.0-28.0)	28.0 (8.0-28.0)	28.0 (-1.0-28.0)	0.687
Organ support-free days	0.0 (-1.0-16.0)	0.0 (0.0-15.0)	0.0 (-1.0-17.0)	0.644

BSI: Bloodstream Infection; CRAB: Carbapenem-Resistant *Acinetobacter baumannii*; CRRT: Continuous Renal Replacement Therapy; HAP: Hospital-Acquired Pneumonia; ICU: Intensive Care Unit; PDR: Pandrug-resistant; VAP: Ventilator-Associated Pneumonia; vHAP: ventilated HAP

Figure S2. Survival curves in the matched cohort (N = 76) according to resistance phenotype. Log-rank p = 0.144.

CRAB, Carbapenem-Resistant *Acinetobacter baumannii*; PDR, Pandrug-resistant

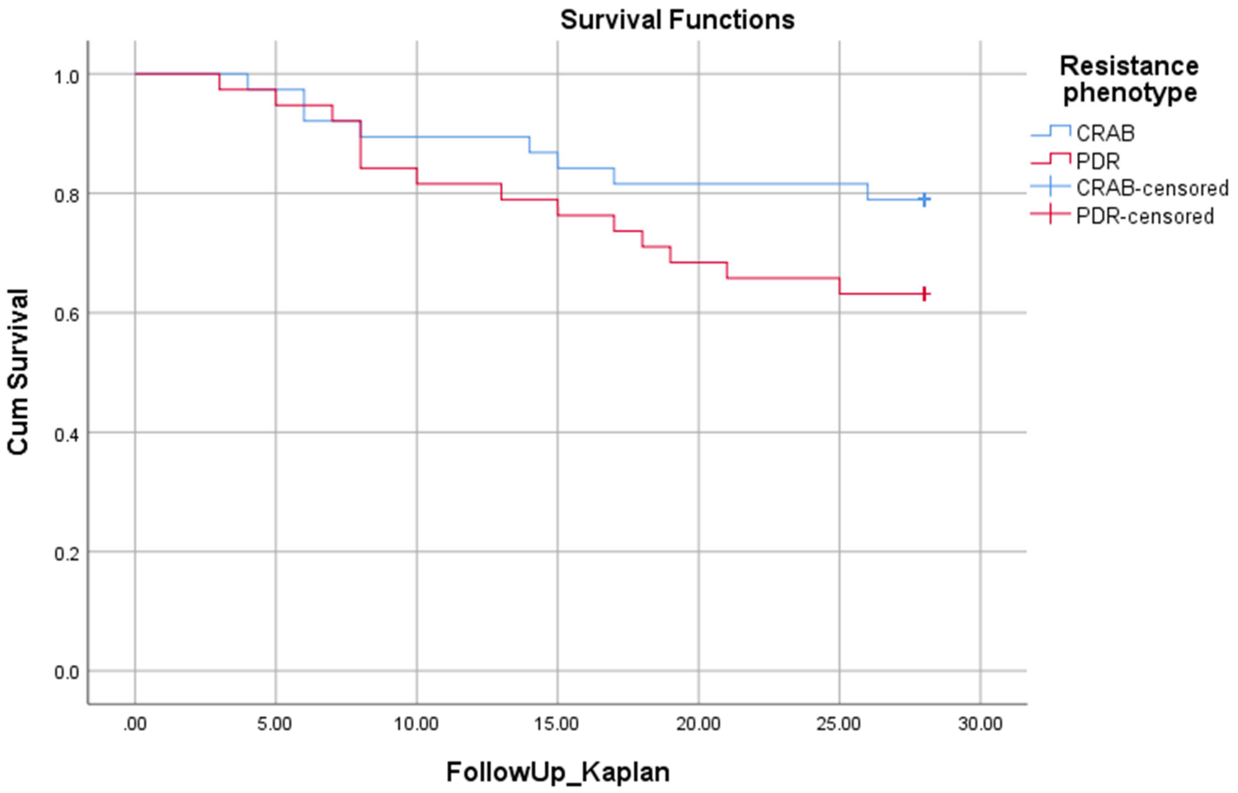

Supplement: Supplementary file 1 [file antibiotics-15-00809-s001.zip › antibiotics-4485525-supplementary.pdf]
